# Natural and synthetic chemicals acting as enhancers of bacterial pathogenic success within the human host

**DOI:** 10.3389/fcimb.2026.1861295

**Published:** 2026-07-08

**Authors:** Maria Escobar-Salom, Isabel M. Barceló, Elena Jordana-Lluch, Jordi Sansó-Sastre, Cristina Lasarte-Monterrubio, Alejandro Beceiro, Antonio Oliver, Carlos Juan

**Affiliations:** 1 Antibiotic Resistance and Pathogenicity of Bacterial Infections Research Group (ARPBIG) group, Health Research Institute of the Balearic Islands (IdISBa), Palma, Spain; 2 Microbiology Department, University Hospital Son Espases (HUSE), Palma, Spain; 3 Centro de Investigación Biomédica en Red, Área Enfermedades Infecciosas (CIBERINFEC), Instituto de Salud Carlos III (ISCIII), Madrid, Spain; 4 Servicio de Microbiología and Instituto de Investigación Biomédica A Coruña (INIBIC), Complexo Hospitalario Universitario A Coruña (CHUAC), A Coruña, Spain

**Keywords:** biofilm, endocrine disruptors, hormones, inter-kingdom signaling, microbial endocrinology, neurotransmitters, pharmaceuticals, virulence

## Abstract

Like any other organism, bacteria are endowed with a myriad of receptors and signaling/regulatory mechanisms to detect chemical stimuli and develop appropriate responses. Whereas several of these chemicals – produced by bacteria themselves – display well-known effects in modulating their virulence-related performance (i.e. quorum sensing signals), many other substances not of microbial but different origin show an ability to influence bacterial behavior. The human organism poses the niche where pathogenic bacteria encounter some of these chemicals, since they are either produced naturally in the body (e.g. hormones, neurotransmitters, etc.) or are synthetic drugs/pollutants (sometimes called xenobiotics) to which people are frequently exposed at relevant doses mostly through inhalation or the diet. Alarmingly, several of these substances display effects that promote bacterial virulence, potentially having obvious negative consequences for the patient. In this scenario, this review integrates and discusses the most notorious data as to how different endogenous and synthetic compounds may impact bacteria biology within the human body context, finally improving their pathogenic success. Delving into this field is needed in order to emphasize the existence of chemicals to which exposure should be avoided as much as possible, and of some host/pathogen pathways that could be targeted so as to curb bacterial pathogenic power and thus alleviate the severity of certain infections.

## 1 Introduction

The idea of living organism-produced soluble/volatile substances acting as triggers for responses not only within a closely related group, but also between very different beings, is not new. This concept –known as inter-kingdom signaling– has been around for decades (Lyte and Ernst, 1992; Hooper and Gordon, 2001; Sperandio, 2004; Hughes and Sperandio, 2008; Kendall and Sperandio, 2016; Irazoki et al., 2019; Boukerb et al., 2021; Wu et al., 2025). This idea was traditionally explored much more from the perspective of microorganism-proceeding molecules that exert different effects on animals, with the obvious example of multiple mediators/virulence factors causing the activation of inflammatory and immune responses after interacting with specific receptors in host cells, as well as other harmful impacts (Li et al., 2026). Additionally, other particular effects of bacterial products on the host have been described, i.e. modulation of energy balance, blood pressure, gut motility, etc (Wu et al., 2025).

Nevertheless, an opposing idea, i.e. chemicals from other kingdoms of life being sensed by bacteria and affecting their performance is also increasingly explored, mostly from the virulence modulation perspective. In this case, the concept of “microbial endocrinology” has often been used, especially for animal hormones/neurotransmitters impacting bacterial behavior within the host (Lyte and Ernst, 1992; Lyte, 2016; Yong et al., 2018). Further, in a kind of analogous, yet opposing, conception to that of drug repurposing (i.e. pharmaceuticals not intended to treat infections displaying unsuspected antibacterial potentials), one could argue that synthetic compounds present in nature as contaminants and acting as modulators of virulence could exist to a greater extent than anticipated. Related to all these facts, here we focus on phenomena of virulence-activating chemical mediators that bacteria come into contact with, not in the general context of the environment, but within the human body. We consider the human host as the niche in which the starring actors of this review encounter: pathogenic bacteria vs natural/synthetic chemicals that influence the performance of the microorganism, potentially aggravating infection outcomes.

While it is true that some of the mediators reviewed here show effects on the human host that could contribute to infection development (e.g., altering the characteristics of the epithelium, dampening the immune response, etc.) (Watters et al., 2014; Kidd et al., 2016; Shen et al., 2024; Koder and Topçakar, 2025; Barceló et al., 2025), this manuscript focuses on the effects impacting the biology of the bacterium itself, ultimately increasing its success for infection. This output is typically mediated by improvements in virulence-related features (growth, biofilm formation, invasion, intracellular survival, toxin release, resistance to killing by immune resources, etc.). However, we will also show cases where specific attenuations occur, which may be beneficial for the bacterium in certain instances, i.e. enabling a stealth pathogen behavior that favors the onset of infection through the generation of a weak alarm/immune response (Sá-Pessoa et al., 2025). Considering these facts and for practical reasons, along the manuscript we will often use the *sensu lato* concept “pathogenic success”. It will allow encompassing the two types of results (increase in the values of virulence parameters vs attenuations) since both outcomes may be advantageous for infection depending on the context.

Many studies about chemical mediators with virulence-activation properties detectable within the human host have been published, albeit from different perspectives and expertise profiles (Boukerb et al., 2021; Wu et al., 2025). In this regard, the most differential point is the nature of the chemicals, i.e. produced by the host (neurotransmitters, hormones, cytokines, etc.) or acquired exogenously (mostly through ingestion or inhalation). In the latter case they are predominantly pharmaceutical products, food additives or synthetic pollutants, sometimes collectively known as xenobiotics (Ilbäck and Friman, 2007). Whatever their origin, these chemicals share the common traits of being found within the body at potentially relevant concentrations, and of being able to activate the bacterial pathogenic success, facts posing the common thread of this review.

Thus, although there are reviews that focus on a certain group of chemicals, this is the first to jointly approach both natural and artificial mediators found in the human body niche showing virulence-boosting effects. The gathered studies are dissected regarding the effects of the different compounds on virulence, species affected, and bacterial receptors/pathways involved when information is available. Moreover, the knowledge gaps present on the different topics approached are also identified and discussed, as well as the potential therapeutic strategies that could be developed from the evidence reviewed.

**Figure 1:**
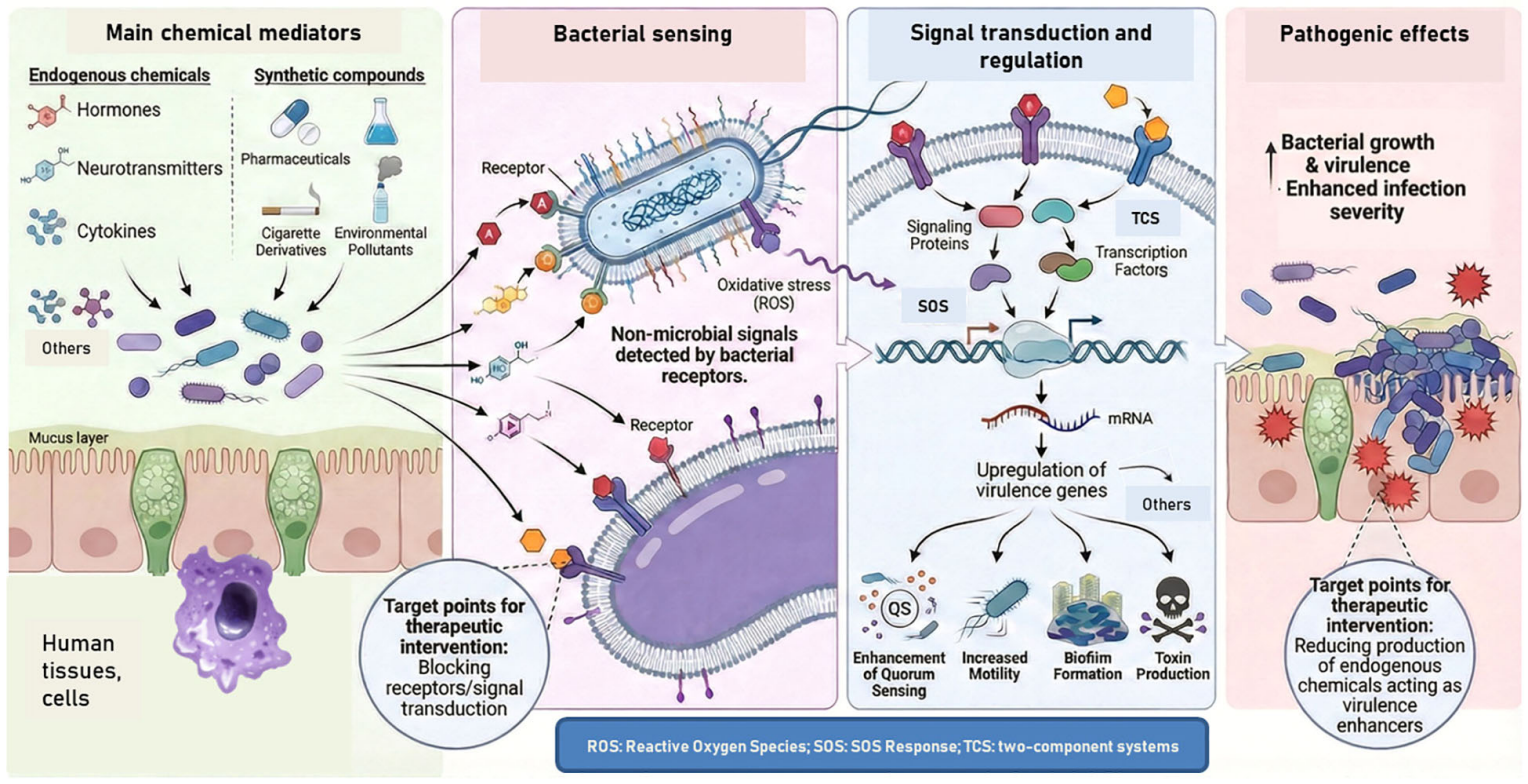
Overview of the research field reviewed in this manuscript, with the main aspects addressed: principal chemical endogenous/exogenous mediators, receptors and bacterial mechanisms involved, consequences for pathogenesis, and potential therapeutic targets/strategies.

In summary, this review covers the need to update and integrate the most relevant data showing that there are natural/synthetic compounds that do not only interact with bacteria within the human body, but also cause a direct modulation of their virulence. This phenomenon could have relevant clinical implications by influencing the evolution of infections. Thus, this review evinces the existence of infection-related risks associated with certain pharmaceuticals or exposure to diverse synthetic chemicals, and also the possibility of related innovative therapeutic strategies: targeting host pathways to reduce the production of endogenous mediators that boost virulence, or blocking the bacterial receptors/pathways that enable the aforementioned chemical-mediated increase in pathogenic success. An overview of the reviewed research field, separating its main aspects is shown in **Figure 1**.

## 2 Endogenous mediators

To address the analysis of such a broad field, data relating to virulence modulating mediators encountered by bacteria within the human host have been divided between endogenously produced chemicals and those acquired exogenously after voluntary or involuntary exposure. To start with, out of all the endogenous compounds displaying enhancing effects on bacterial pathogenic success, hormones are the most explored. Accordingly, an overview of the most relevant published information dealing with the impacts of these mediators on bacterial virulence is shown in **Table 1**, splitting them into groups with a larger number of studies (sex vs stress hormones/catecholamines). The rest of endogenous human chemicals are revised next (**Table 2**). In order to provide complementary perspectives of the topic, it is approached in the text by organizing the information mostly according to the pathogen, whereas in tables data are primarily organized based on each group/specific chemicals analyzed. In these two tables and later also in **Table 3** (synthetic chemicals), the columns specifying: i) main effects reported and *in vitro* and/or *in vivo* models used; ii) number/nature of bacterial strains included, and iii) level of evidence/assays supporting the identification of underlying mechanisms, will help the reader to quantify ​​the robustness of each study and the potential real applicability of the derived data.

**Table 1:** Overview of the main published data regarding the virulence-boosting capacities of human sex and stress hormones on relevant bacterial pathogens.

General group of mediators	Specific mediators	Species affected	Main effects/models used	Concentrations of chemical mediators that caused effects^a^	Strains used	Bacterial receptors, pathways or mechanisms involved	Evidence/assays to determine underlying mechanisms	Study
Sex hormones and related compounds	Estradiol, estriol	*P. aeruginosa*	Acquisition of mucoid phenotype, increased biofilm formation	Physiological	PAO1 type strain and CF –proceeding isolates	ND	ND	Chotirmall et al., 2012
	Estriol, testosterone		Increased biofilm formation, motility, and rhamnolipid and elastase production. Increased release of OMVs. Increased virulence in murine model of infection	Physiological and supra- physiological	PAO1 type strain and various KO mutants	Bacterial envelope stress caused by the hormones’ polar nature, sensed by surface signaling systems including the *muc* operon	Transcriptomics, use of KO mutants, membrane fluidity photobleaching studies	Vidaillac et al., 2020
	Estradiol	*P. gingivalis*	Increased growth rate, biofilm formation, and invasion of gingival epithelial cells. Reduction in the intrinsic inflammatory power of *P. gingivalis* cells	pre- and post-menopausal physiological concentrations	W50 clinical isolate	ND	ND	Demirel et al., 2022
		UPEC	Increased expression of virulence genes (*usp, sfa/foc, cnf1*), invasion of bladder epithelial cells. Decreased elicited inflammatory response and attenuated virulence in *C. elegans*	pre- and post-menopausal physiological concentrations	CFT073, C7, and C149 clinical strains	ND	ND	Engelsöy et al., 2021; Gümüş et al., 2019
		*P. aeruginosa*	Increased biofilm formation, pyocianin production, and motility	Supra- physiological	PAO1 and ATCC27853 type strains and CF isolates	ND	ND	Tyrrell and Harvey, 2020
	Testosterone	UPEC	Increased growth rate, biofilm formation, invasion of bladder epithelial cells, endotoxin release, and general virulence in *C. elegans* model	Physiological	CFT073 clinical strain	ND	ND	Wu et al., 2024
		*S. aureus*	Increased general virulence in murine model of skin infection. Increased hemolytic activity, cytotoxicity, and oxidative stress resistance. Upregulation of genes encoding α-hemolysin, phenol-soluble modulin, and accessory gene regulator quorum-sensing components (*agrC, agrA, agrB, agrD*)	Physiological and supra- physiological	Newman reference strain and several MRSA clinical isolates	AgrC membrane receptor	Molecular docking simulations and use of an *agcR* KO mutant	Luo et al., 2024
	Corticosteroids (fluticasone and budesonide)	*P. aeruginosa*	Increased biofilm formation, decreased cytotoxicity and invasion of A549 alveolar epithelial cells. Reduction in the intrinsic inflammatory power of *P. aeruginosa* cells	Physiological: attainable after treatment with corticosteroids	PAO1 and PA14 reference strains	Alterations in membrane fluidity, activation of *muc* operon	Transcriptomics, commercial membrane fluidity labelling solutions	Jordana-Lluch et al., 2025
Stress-related hormones, catecholamines, and catecholamine inotropes	Norepinephrine, epinephrine, dopamine, and serotonin	*P. aeruginosa*	Chemotaxis	Physiological (for dopamine in specific locations: kidney, colon, carotid body, etc.) and supra- physiological	PAO1 reference strain	TlpQ acting as receptor	KO mutants. Purification of TlpQ and Micro-calorimetric titrations of TlpQ‐LBD with each ligand	Monteagudo-Cascales et al., 2025;
	Norepinephrine, epinephrine, dopamine and L-DOPA	*P. aeruginosa*	Increased growth	Physiological (for dopamine and L-DOPA in specific locations/after treatment) and supra-physiological	PAO1 reference strain	TonB-dependent iron transporters PirA and PiuA	Use of PirA/PiuA KO mutants. Proteomic and qRT-PCR approaches. Kinetics of 55Fe accumulation in *P. aeruginosa* cells.	Perraud et al., 2022
	Norepinephrine, epinephrine	*P. aeruginosa*	Increased growth, biofilm formation, expression of genes involved in the production of pyocyanin, *pslA* (essential for the biosynthesis of exopolysaccharides), and other virulence related genes (*exoS, exoT, toxA*, and *aprA*)	Supra- physiological	PAO1 reference strain	ND	ND	Medina-Lopez et al., 2022
		ETEC	Increased biofilm formation and upregulation of the virulence genes *feaG, estA, estB,* and *elt*	Supra- physiological	CVCC230 reference strain and 9 ETEC isolates from veterinary samples	ND	ND	Niu et al., 2023
		*P. gingivalis*	Increased expression of genes related to iron acquisition (*hmuR*), oxidative stress resistance (*tpx, oxyR, dps, sodB,* and *aphC*), and pathogenesis (*hem, hagA* and *ragA*)	Supra- physiological	W83 reference strain	ND	ND	Graziano et al., 2013
		*C. acnes*	Increased biofilm formation	Supra- physiological	Acneic strain HL045PA1/HM-516 and non acneic strain HL110PA3/HM-554	ND	ND	Borrel et al., 2019
	Norepinephrine, epinephrine, and serotonin	*E. faecalis, E. faecium*	Increased biofilm formation and boosted adhesion to Caco-2/TC7 intestinal cells and HaCaT keratinocytes	Physiological and supra-physiological	*E. faecalis* pathogenic and probiotic strains; *E. faecium* SF68 type strain	VicK acting as receptor	In silico structure modeling and molecular docking	Cambronel et al., 2020; Scardaci et al., 2022
	Norepinephrine	*P. aeruginosa*	Increased growth rate, production of pyocianin and elastase, motility, and invasion of epithelial cells	Physiological (gastrointestinal tract)	PA14 reference strain	*las* quorum-sensing pathway	Transcriptome analysis; promoter::lacZ reporter fusion and β-galactosidase activity	Hegde et al., 2009
			Increased adherence to contact lenses, biofilm formation, and virulence in a keratitis murine model	Supra- physiological	ATCC 19660 reference strain	ND	ND	Li et al., 2020
		UPEC	increased biofilm formation, adhesion, motility, and metabolic activity	Physiological (urine)	12 UPEC clinical strains	ND	ND	Ignatova et al., 2022
		*V. parahemolythicus; V. mimicus*	Increased growth and cytotoxicity towards Caco-2 cells	Supra- physiological	*V. parahemolyticus* RIMD2210633, RIMD2210876 and RIMD2211709; *V. mimicus* RIMD2218070 reference strains	ND	ND	Nakano et al., 2006; Nakano et al., 2007
		*A. baumannii*	Increased growth, adhesion, and biofilm formation	Physological after treatment with dopamine 3 µg/kg/min (typical dosage for ICU/shock patients)	ATCC 17978 reference strain and 25 MDR clinical isolates	ND	ND	Inaba et al., 2016
		*A. hydrophyla*	Increased protease activity and virulence in a fish infection model	Supra- physiological	Five strains from diseased carps	ND	ND	Gao et al., 2019
		*S. pneumoniae*	Increased growth, biofilm formation, and expression of virulence factors such as neuraminidase	Physological (following inotrope administration)	TIGR4 and D39 reference strains and KO mutants	Surface proteins PspA and PspC, and PiuA iron transporter	Use of *pspA* and *pspC* KO mutants in catecholamine inotrope responsiveness *in vitro* assays. Purification of PiuA protein, *in vitro* experiments of iron binding, NMR spectroscopy	Sandrini et al., 2014; Zhang et al., 2020
	Epinephrine	*P. aeruginosa*	Increased biofilm formation and virulence towards *G. mellonella*	Physiological and supra-physiological	H103 reference strain (PAO1 subline)	ND	ND	Cambronel et al., 2019
		*M. luteus*	Increased biofilm formation, in which the amount of polysaccharides and extracellular DNA were consistently increased	Physiological	C01 (a skin colonizer strain from healthy volunteer)	ND	ND	Gannesen et al., 2021; Gannesen et al., 2022
		*M. tuberculosis*	Increased growth and biofilm formation	Supra-physiological	H37Ra reference strain	MprAB two-component system	Use of *mprB* knock down/*mprB* overexpressing mutants. Molecular docking	Lei et al., 2023
	Catecholamine inotropes	*P. aeruginosa*	Increased biofilm formation on endotracheal tubes, production of pyoverdine, and toxicity towards respiratory epithelium	Physiological: attainable after treatment with dopamine.	PA14 reference strain and a clinical isolate	ND	ND	Freestone et al., 2012
		*S. epidermidis*	Increased adhesion and biofilm formation	Physiological: attainable after treatment with norepinephrine or dobutamine	RP62A reference strain and three clinical isolates	ND	ND	Lyte et al., 2003
	Cortisol	*P. gingivalis*	Increased growth	Physiological and supra-physiological	ATCC 33277 reference strain and five clinical isolates	Capture of ROS by cortisol in initial growth stages?	Not experimentally verified. Hypothesis from the authors	Akcalı et al., 2014; Klomp et al., 2023
	Dopamine	*P. aeruginosa*	Increased biofilm formation, motility, and production of alginate, rhamnolipids, proteases, and pyocyanin	Physiological (brain) and supra-physiological	PAO1 reference strain	Dopamine itself acting as a quorum sensing-like signal?	Not experimentally verified. Hypothesis from the authors	Xiang et al., 2024
		*S. Typhimurium*	Increased growth and virulence in murine model of infection	Physiological (brain) and supra-physiological; Physiological in serum after treatment with IP dopamine in murine model	14028 reference strain and KO mutants	Sensor kinase QseC and iron transporters SitABCD	Use of *qscE/sitABC* KO mutants in murine models of infection, *in vitro* growth, iron uptake measurements, qRT-PCR	Dichtl et al., 2019
		*S. lugdunensis*	Increased biofilm formation and boosted detachment of cells from preformed biofilms when adding heparin	Physiological (brain and after intravenous administration) and supra-physiological	15 clinical strains	ND	ND	Frank and Patel, 2008
	CRH	*S. pneumoniae*	Increased growth, production of capsule and pneumolysin, and virulence in murine model	Physiological	ATCC 6301 reference strain	ND	ND	Ndjom and Jones, 2015; Ngo Ndjom et al., 2017

a Considering typical concentrations previously reported in serum in regular situations (Steckl and Ray, 2018) unless otherwise specified for particular contexts (Thompson et al., 1999). ND, no data; CF, cystic fibrosis; OMV, outer membrane vesicle; UPEC, uropathogenic *E. coli*; ETEC, enterotoxigenic *E. coli*; ROS, reactive oxygen species; MRSA, methicillin-resistant *S. aureus*; LBD, ligand binding domain; MDR, multi-drug resistant; NMR, nuclear magnetic resonance; CRH, corticotropin-releasing hormone; IP, intra-peritoneal.

**Table 2:** Overview of the main published data regarding the virulence-boosting capacities of natriuretic peptides, neurotransmitters, cytokines, and other human endogenous mediators on relevant bacterial pathogens.

General group of mediators	Specific mediators	Species affected	Main effects/models used	Concentrations of chemical mediators that caused effects^a^	Strains used	Bacterial receptors, pathways or mechanisms involved	Evidence/assays to determine underlying mechanisms	Study
Natriuretic peptides	ANP, CNP	*C. acnes*	Increased competitiveness of *C. acnes* towards *S. aureus* in mixed biofilms	Physiological	*S. aureus* MFP03 reference strain. C. acnes HL045PA1 and HL043PA2 acneic isolates	ND	ND	Gannesen et al., 2018
	BNP, CNP	*P. aeruginosa, P. fluorescens*	Increased glial cell necrosis and modifications in LPS biosynthesis	Physiological (tissues)	*P. aeruginosa* PAO1 reference strain. *P. fluorescens* MF37 reference strain	cAMP-binding protein Vfr	Use of a *vfr* KO mutant in cytotoxicity assays (*P. aeruginosa*)	Veron et al., 2007; Veron et al., 2008
	ANP	*C. acnes*	Boosted ratio of *C. acnes* biomass in the dual biofilm with *S. epidermidis*; increased metabolic activity of cells within biofilms	Physiological	*S. epidermidis* ATCC14990 reference strain, *C. acnes* HL043PA2 acneic strain	ND	ND	Ovcharova et al., 2021
		*S. aureus, K. schroeteri*	Increased biofilm formation on hydrophilic surfaces like glass	Supra-physiological	*K. schroeteri* H01 skin isolate from a healthy volunteer; *S. aureus* ATCC 6538P reference strain	ND	ND	Diuvenji et al., 2023
	BNP	*M. luteus, A. faecalis*	Increased tolerance to certain types of stress (e.g. H2O2, antibiotics, salinity, heat, and pH shock)	Physiological and supra- physiological	M. luteus C01 and A. faecalis DOS7 reference strains	ND	ND	Loiko et al., 2022
	CNP	*C. acnes*	Increased proliferation of *C. acnes*, its competitive properties against *S. epidermidis*, and metabolic activity of the biofilm	Physiological and supra- physiological	*S. epidermidis* ATCC14990 reference strain, *C. acnes* ATCC HM-514 acneic strain	Competition for zinc?	RNA-seq: upregulation of the zinc ABC transporter in *C. acnes.*	Ovcharova et al., 2023
		*P. aeruginosa*	Increased capacity to kill *C. elegans*, increased production of AHL, HCN, and exotoxin A. Decreased biofilm formation	Supra- physiological	PAO1 reference strain and different KO mutants	Vfr, PtxR regulator	Use of *vfr/ptxR* KO mutants in *C. elegans* model and exotoxin A production assays	Blier et al., 2011
Neurotransmitters	Substance P	*P. fluorescens*	Increased swarming motility and damages (permeability) and release of IL-8 in infected Caco-2/TC7 cells	Physiological (synaptic cleft)	MFN1032 reference strain	ND	ND	Biaggini et al., 2015
		*S. epidermidis, S. aureus*	*S. epidermidis*: increased biofilm formation *S. aureus*: increased production of Enterotoxin C2 Both: boosted adhesion potential on keratinocytes	Physiological (synaptic cleft)	*S. aureus* MFP03 and *S. epidermidis* MFP04 skin isolates from healthy volunteers	Ef-Tu (as substance P receptor)	Immunoprecipitation and MALDI-TOF analysis	N'Diaye et al., 2016b
		*B. cereus*	Increased biofilm formation, cytotoxicity, and production of collagenase and superoxide dismutase	Physiological (synaptic cleft)	*B. cereus* MFP01, *S. aureus* MFP03 and *S. epidermidis* MFP04 isolates from healthy volunteers	Ef-Tu (as substance P receptor)	Immunoprecipitation and MALDI-TOF analysis	Mijouin et al., 2013
	CGRP	*S. epidermidis*	Increased adherence to keratinocytes, but reduced internalization and biofilm formation	Supra- physiological	*S. aureus* MFP03 and *S. epidermidis* MFP04 skin isolates from healthy volunteers	DnaK chaperone (as CGRP receptor)	Immunoprecipitation and MS	N'Diaye et al., 2016a
	GABA	*P. aeruginosa*	Increased cyanogenesis, production of oxygen-scavenging proteins, cytotoxicity, and killing capacity in *C. elegans* model	Physiological (CSF, brain, etc.)	PAO1 reference strain	ND	ND	Dagorn et al., 2013a
		*P. aeruginosa, P. putida*	Chemotaxis	Physiological (CSF, brain, etc.)	*P. aeruginosa* PAO1 reference strain; *P. putida* KT2440 reference strain	PctC and McpG acting as receptors	Proteins purification and Isothermal titration calorimetry. Use of a *mcpG* KO mutant in plant root colonization assays	Rico-Jiménez et al., 2013; Reyes-Darias et al., 2015
		*P. fluorescens*	Increased necrotic-like activity on glial cells	Physiological (CSF, brain, etc.)	mf37, Pf0–1 and SBW25 reference strains	ND	ND	Dagorn et al., 2013b
	Dynorphin A (1-17)	*P. aeruginosa*	Increased pyocianin production and killing capacity in *C. elegans* model	Physiological (intestine luminal flushings)	PAO1 reference strain and various KO mutants	Global transcriptional regulator MvfR	Use of a *mvfR* KO mutant in virulence assays against *C. elegans* and probiotic bacteria	Zaborina et al., 2007
	Dynorphin (1-13)		Increased pyocianin production, up-regulation of efflux pumps and *pmrAB* and *arn* operons, involved in the antimicrobial peptide-resistance response by modifying lipid A	Physiological (gut)	PAO1 reference strain	ParRS two-component system	Photoaffinity labeling and mass spectrometry to identify protein interacting partners of dynorphin. Use of a parS KO mutant in pyocianin production assays	Wright et al., 2017
Cytokines	IL1-β	*E. coli*	Increased growth of virulent strains	Physiological (inflamed tissues)	Six virulent and four avirulent *E. coli* strains	ND	ND	Porat et al., 1991
		*A. actinomycetemcomitans*	Increased biofilm formation	Physiological (inflamed tissues)	Four clinical strains and derived KO mutants	Bacterial interleukin receptor I (BilRI)	MS, protein purification, microplate protein-ligand binding assays	Paino et al., 2011; Paino et al., 2013
		*S. aureus*	Increased planktonic growth and biofilm formation	Physiological (inflamed tissues) and supra- physiological	Clinical isolates (number not specified)	ND	ND	Kanangat et al., 2001; McLaughlin and Hoogewerf, 2006
	IL1-β, IL-6	*S. aureus, P. aeruginosa, Acinetobacter* spp.	Increased intracellular growth within monocytes	Physiological (inflamed tissues)	Clinical isolates (number not specified)	ND	ND	Kanangat et al., 1999
	IL1-β, TNF-α, IL-6, IL-8, IFN-γ	*E. coli*	Increased growth and virulence in *C. elegans* model, but reduced biofilm formation and hemolytic activity	Physiological (urine)	CFT073 UPEC clinical strain	ND	ND	Engelsöy et al., 2019
	IL-8	*M. tuberculosis*	Increased entry into human neutrophils	Physiological under infection conditions (tissues and fuids)	H37Rv reference strain and mutants	AtsG, GlmU and SahH receptors	Protein purification, affinity chromatography, liquid chromatography ESI MS-MS and surface plasmon resonance. Use of mutants overproducing the AtsG, SahH or GlmU proteins	Krupa et al., 2015; Dziadek et al., 2016
	IFN-γ	*P. aeruginosa*	Increased expression of the adhesion PA-1 (LecA) lectin	Physiological (inflamed micro-environments)	PAO1 and ATCC 27853 reference strains	OprF porin	Purification of membrane proteins, immunoblotting with IFN-γ anti-body, immunoprecipitation. Use of *oprF* KO mutant.	Wu et al., 2005
		*M. tuberculosis*	Increased respiration and growth in granuloma model, survival within macrophages, and secretion of extracellular vesicles	Physiological (inflamed micro-environments)	H37Rv reference strain and two clinical isolates	MmpL10	Immunoblotting with IFN-γ anti-body, MS, use of *mmpL10* KO mutant. In silico molecular modeling (docking). Proteome analysis	Ahmed et al., 2022; Hop et al., 2024
	TNF-α	*S. flexneri*	Enhanced invasion of HeLa cells and uptake by human and murine macrophages	Supra-physiological	S100 reference strain	ND	ND	Luo et al., 1993
	TNF-α, IL-8	*N. meningitidis*	Enhanced resistance to complement-mediated lysis	IL-8: Physiological (inflamed micro-environments). TNF-α: Supra-physiological	MC58 reference strain and KO mutants	Type IV pili subunits, PilE, and PilQ	Use of *pilQ* and *pilE* KO mutants in murine model. Transmission electron micrographs: use of antibodies conjugated with colloidal gold particles to detect internalized chemokines	Mahdavi et al., 2013
Others	Insulin	*P. aeruginosa*	Increased biofilm formation	Physiological after subcutaneous/IV administration of insulin	PAO1 reference strain	ND	ND	Watters et al., 2013; Watters et al., 2014
	Adenosine		Increased expression of the adhesion PA-1 (LecA) lectin	Physiological under surgical/hypoxic stress	ATCC-27853 reference strain	ND	ND	Kohler et al., 2005; Patel et al., 2007
	Gastrin	*H. pylori*	Increased growth	Physiological luminal and serum levels	Clinical isolates (number not specified)	ND	ND	Chowers et al., 1999
	EGF	*M. tuberculosis, M. avium*	Increased growth *in vitro* and survival within macrophages	Physiological in several fluids (tears, urine, milk, etc.)	*M. tuberculosis* H37Rv reference strain and three *M. avium* clinical strains	GAPDH-like receptor	^125^I-EGF binding assays, Immunoblot, protein purification, Edman chemistry microsequencing	Bermudez et al., 1996

a Considering healthy individuals regular concentrations in serum as a general rule, unless otherwise specified. BNP, Brain Natriuretic Peptide; CNP, C-type Natriuretic Peptide; AHL, acylhomoserine lactone; HCN, hydrogen cyanide, diffusible gas working as a toxic factor against eukaryotic organisms; ND, no data; ANP, Atrial Natriuretic Peptide; CGRP, calcitonin gene-related peptide; Ef-Tu, thermo unstable ribosomal elongation factor; GABA, gamma-aminobutyric acid; MmpL10, mycobacterial membrane protein large 10; EGF, epidermal growth factor; GAPDH, glyceraldehyde-3-phosphate dehydrogenase; IV, intra-venous; CSF, cerebrospinal fluid; MS, mass spectrometry; ESI MS-MS, electron spray ionization tandem mass spectrometry.

**Table 3:** Overview of the main published data regarding the bacterial virulence-boosting capacities of synthetic chemicals to which humans are exposed.

General group of mediators	Specific mediators	Species affected	Main effects/models used	Concentrations of chemical mediators that caused effects^a,b^	Strains used	Bacterial receptors, pathways or mechanisms involved	Evidence/assays to determine underlying mechanisms	Study
Endocrine disruptors	Phthalates	*P. aeruginosa*	Increased biofilm formation	Supra-physiological	H103 reference strain (PAO1 subline)	ND	ND	Louis et al., 2022
			Increased biofilm densification and resistance to chlorine; up-regulation of genes involved in polymeric substance secretion and oxidative stress resistance	Physiological	PAO1 reference strain	ND	ND	Wang et al., 2022a
		*L. pneumophila*	Increased motility and resistance to rifampin and levofloxacin	Physiological	CIP107629 (Paris) reference strain	ND	ND	Crépin et al., 2023
		*H. pylori*	Increased cytotoxicity towards gastric epithelial cells	Supra-physiological	ATCC 43054 reference strain	ND	ND	Lin et al., 2013
	Bisphenol A, phthalates, parabens, and triclosan	*P. aeruginosa*	Increased biofilm formation and adhesion to lung cells	Physiological after exposure of individuals and relevant in environmental waters	H103 reference strain (PAO1 subline)	ND	ND	Thiroux et al., 2023b
	Parabens	*A. calcoaceticus, S. maltophilia*	Increased cell viability, density, and thickness of biofilms	Physiological and relevant in drinking waters	Environmental strains isolated from drinking tap water	ND	ND	Pereira et al., 2023
	Methylparaben	*S. maltophilia*	Increased swimming motility and production of protease and gellatinase	Physiological and relevant in drinking waters	Environmental strain isolated from drinking tap water	ND	ND	Pereira et al., 2023
	Bisphenol A	*S. mutans*	Increased resistance to hydrogen peroxide	Physiological into oral cavity and dental biofilms	UA159 reference strain	ND	ND	Kim et al., 2019
	Triclosan		Increased biofilm formation and adhesion to gingival epithelial cells. Up-regulation of genes related to adherence and biofilm formation such as *comD, gtfC,* and *luxS*	Physiological (saliva) after usage of triclosan- containing oral hygiene products	ATCC 25175 and ATCC 35668 reference strains	ND	ND	Bedran et al., 2014
		*S. aureus*	Increased binding to collagen, fibronectin and keratin, and plastic/glass surfaces. Boosted nasal colonization in rat model	Physiological (nasal secretions)	SH1000 reference strain	ND	ND	Syed et al., 2014
Opioids	Morphine	*P. aeruginosa*	Chemotaxis. Increased mortality in a murine model of gut-derived sepsis. Improved mucus production-supressing and epithelial barrier-disrupting activities	Supra-physiological	PAO1 and ATCC 27853 reference strains	ND	ND	Babrowski et al., 2012
		*E. faecalis*	Increased adhesiveness and collagenase production, increased virulence in rat model of anastomotic surgery	Supra-physiological (*in vitro* experiments), physiological in animal model (chronic morphine administration)	Four strains isolated from anastomotic tissues	ND	ND	Shakhsheer et al., 2016
	U-50488	*P. aeruginosa*	Increased production of pyocianin and pyoverdin, and virulence towards *C. elegans*	Not approved for human use. ND	PAO1 reference strain	ND	ND	Zaborin et al., 2012
Benzodiazepines	Diazepam, midazolam	*P. aeruginosa*	Increased biofilm formation on plastic plates and endotracheal tubes. Decreased cytotoxicity and invasion of A549 alveolar epithelial cells. Reduction in the intrinsic inflammatory power of *P. aeruginosa* cells	Physiological after administration of high doses of benzodiazepines	PAO1 and PA14 reference strains	ND	ND	Barceló et al., 2025
	PK 11195	*P. fluorescens*	Increased biofilm formation, boosted adhesion to glial cells and glass surfaces	Supra-physiological	MF37 reference strain and 5 clinical isolates	bactTSPO	In-silico analysis, membrane purification, radioligand binding studies, WB, ESI MS-MS	Chapalain et al., 2009
Tobacco smoke	Not specified	P. aeruginosa	Increased biofilm formation; up-regulation of genes related to biofilm (*pilF, flgK, algC, lasI*)	Direct exposure	PAO1 reference strain	ND	ND	Antunes et al., 2012
			Increased biofilm formation, resistance to neutrophil killing, mortality in murine model, and up-regulation of efflux pumps. Improved levofloxacin resistance phenotype	Cigarette smoke extract added to the culture/assay media	PAO1 reference strain	Oxidative stress -protective responses (e.g. SOS DNA repair mechanism)	Increased expression of thiol peroxidase	Chien et al., 2020
		*P. gingivalis*	Increased monospecies and dual species (plus *S. gordonii*) biofilm formation and translation of major fimbrial antigen (FimA)	Cigarette smoke extract added to the culture/assay media, including physiologically relevant doses of nicotine	*P. gingivalis* W83 and ATCC 33277 reference strains. *S. gordonii* DL1 reference strain	ND	ND	Bagaitkar et al., 2010; Bagaitkar et al., 2011
		*S. mutans, S. sanguis*	Increased colony growth, adhesion, and biofilm formation	Direct exposure/cigarette smoke concentrate added to the culture/assay media	*S. mutans* ATCC 25175 and *S. sanguis* ATCC 10556 reference strains	ND	ND	Zonuz et al., 2008; Baboni et al., 2010
		*S. pneumoniae*	Increased biofilm formation; up-regulation of genes encoding for detoxification enzymes, stress resistance proteins, osmoregulator transporters, and efflux pumps. Attenuation of the pore-forming interactions of pneumolysin	Cigarette smoke condensate/extract added to the used media	EF3030 and PMP1287 reference strains and five clinical isolates	Two component regulatory system 11, Oxidative stress and SOS responses	Upregulation of TCS 11 and other genes related with de-toxification of glyoxals and aldehydes for instance	Mutepe et al., 2013; Cockeran et al., 2014; Manna et al., 2018; Cockeran et al., 2020
		*S. aureus*	Increased biofilm formation, binding to immobilized fibronectin and A549 cells, transcriptional induction of oxidoreductases devoted to counteracting ROS toxicity; up-regulation of *fnbA* (fibronectin binding protein A)	Cigarette smoke extract added to the used media	RN6390, Newman, USA300 (MRSA), and 502A reference strains	Oxidative stress responses, down-regulation of *agr* system, involved in biofilm dispersal	Upregulation of oxidoreductase genes related with de-toxification of ROS	Kulkarni et al., 2012
		*S. aureus*	Increased biofilm formation, invasion, and persistence within A549 bronchial-alveolar epithelial cells	Cigarette smoke extract added to the used media	SH1000 and Newman reference strains, KO mutants and several MRSA clinical strains	Oxidative stress and SOS SOS mutagenic DNA-repair pathway, RexAB two-components system. Selection of mutations linked to persistent infection	Increased mutation frequency, use of KO mutants in *rexAB* (involved in DNA recombinational repair)	Lacoma et al., 2019
		*S. aureus (MRSA)*	Increased adhesion and invasion of HaCaT cells, increased resistance to macrophage and neutrophil killing and to the effects of antimicrobial peptides, increased virulence in murine models of pneumonia	Cigarette smoke extract added to the used media	Newman and USA300 MRSA reference strains	ND	ND	McEachern et al., 2015; Kulkarni et al., 2016
		*S. aureus (MRSA)*	Up-regulation of genes encoding for adhesins (clumping factor B and fibronectin- and fibrinogen-binding proteins A and B), staphylococcal protein A, staphylocoagulase, and nuclease	Cigarette smoke extract added to the used media	Newman and USA300 MRSA reference strains	ND	ND	Kulkarni et al., 2016
		*P. aeruginosa, S. aureus, K. pneumoniae, K. oxytoca, S. pneumoniae, S. marcescens,* and *P. mirabilis* from chronic rhinosinusitis patients	Biofilm produced to a greater extent proportional to two factors: the patient being a smoker, and length of exposure to smoke *in vitro*	Direct exposure	PAO1 (*P. aeruginosa*) and 29213 (*S. aureus*) reference strains, and clinical isolates from mucopurulent sinonasal secretions	ND	ND	Goldstein-Daruech et al., 2011
Nicotine		*P. gingivalis*	Increased production of proteins involved in metabolism, virulence, and acquisition of peptides, protein synthesis and folding, transcription, and oxidative stress.	Physiological (saliva, smoking individuals)	W83 reference strain	ND	ND	Cogo et al., 2012
		*L. casei, E. faecalis, A. viscosus* and *R. dentocariosa*	Increased biofilm formation and planktonic growth	Physiological (saliva, smoking individuals)	*L. casei* ATCC 393, *R. dentocariosa* ATCC 17931, *E. faecalis* ATCC 29212, and *A.* *viscosus* ATCC 43146 reference strains	ND	ND	DuBois et al., 2014
		*S. mutans*	Increased formation, thickness, and cell viability in biofilms (both monospecific and in dual assays with *S. sanguinis*); increased production of extracellular polysaccharides and metabolic activity, acquisition of more spherical shape by bacterial cells	Physiological (saliva, smoking individuals)	S. mutans ATCC 700610, ATCC 700611 and ATCC 25175 reference strains and four clinical isolates; S. sanguinis ATCC 10556 referece strain	ND	ND	Huang et al., 2012; Li et al., 2014; Huang et al., 2014; Huang et al., 2015
			Increased expression of the virulence-related ldh and the phosphotransferase system (PTS)-associated genes and of lactate production	Physiological (saliva, smoking individuals)	ATCC 700610 reference strain	ND	ND	Li et al., 2016
		*S. aureus*	Increase in attachment, extracellular DNA release, and biofilm formation. Impaired invasion of A549 alveolar epithelial cells and decreased expression of several virulence factor-encoding genes	Physiological (saliva, smoking individuals)	USA300 FPR3757 MRSA strain	ND	ND	Shi et al., 2019
		*M. tuberculosis*	Increased intracellular and extracellular growth; up-regulation of virulence factors and genes related to resistance to antimicrobial peptides	Physiological (serum, smoking individuals)	H37Rv reference strain	ND	ND	de Haro-Acosta et al., 2021; Rivas-Santiago et al., 2023
E-cigarette vapor	Not specified	*S. mutans*	Increased adhesion, biofilm formation, and planktonic growth	Direct exposure	ATCC 25175 reference strain	ND	ND	Rouabhia and Semlali, 2021
		*S. pneumoniae*	Nicotine-dependent increased biofilm formation	E-cigarette vapor extract added to the used media	TIGR4 reference strain	Stress induced by free radicals and reactive oxygen species	RNA-seq; up-regulation of genes predominantly involved in metabolism and stress response	Bagale et al., 2020
		*S. aureus (MRSA)*	Increased biofilm formation, adherence, and invasion of epithelial cells, resistance to antimicrobial peptide LL-37, and hyper-expression of virulence genes, translated into more severe outcomes in murine model of pneumonia	Direct exposure and E-cigarette vapor extract added to the used media	Not specified MRSA strain	ND	ND	Hwang et al., 2016
	Comparative study: e-cigarette vapor vs tobacco smoke	*H. influenzae, S. pneumoniae, S. aureus* and *P. aeruginosa*	Increased biofilm formation upon exposure to both stimuli; *G. mellonella* larvae survival decreased after infection with all the tested pathogens previously exposed to both vapor and smoke	Cigarette smoke vs E-cigarette vapor extracts added to the used media	*H. influenzae* ATCC 49766, *S. aureus* ATCC 29213, *S. pneumoniae* ATCC 49619 and *P. aeruginosa* ATCC 27853 reference strains	ND	ND	Gilpin et al., 2019
Others	Paracetamol	*S. aureus*	Increased biofilm formation, increased accumulation of N-acetyl-glucosamine-containing components of the biofilm matrix	Physiological after oral administration of paracetamol	Newman, 8325–4 and RN6390 reference strains and several clinical and commensal isolates	ND	ND	Sultan et al., 2021
	PFAS		Increased virulence in *C. elegans* model, mediated by hyper-expression of the virulence gene regulator *saeR* and *hla* (α-hemolysin)	Physiological after exposure of individuals and relevant in environmental waters	Newman reference strain	ND	ND	Mangu et al., 2022
	Various drugs		Increased biofilm formation on plastic and respiratory/duodenal probes upon exposure to lovastatin, loratadine, diclofenac, and verapamil, among others	Physiological after administration of drugs	Two clinical isolates	ND	ND	Carmona-Orozco and Echeverri, 2024

a Considering the drugs/pollutants typical concentrations previously reported in serum in regular situations, unless otherwise specified for particular contexts.^b^In the case of tobacco smoke/e-cigarette vapor, two approximations have been followed in the literature: direct exposure to vapor/smoke in special chambers vs addition of smoke/vapor extracts to the culture/assay media. They are both generally accepted to be good approximations to the real exposure of colonizing bacteria to smoke/vapor in the airways (Downs et al., 2011). ND, no data;ROS, reactive oxygen species; PFAS, Per- and Poly-fluoroalkyl substances; MRSA, methicillin-resistant S. aureus; WB, western blot; ESI MS-MS, electron spray ionization tandem mass spectrometry; TCS, two components system.

### 2.1 Sex hormones

*Pseudomonas aeruginosa* is one of the most important opportunistic pathogens, showing an outstanding capacity for antibiotic resistance development. It causes a great variety of infections, from acute nosocomial to chronic pulmonary in patients with underlying respiratory diseases such as cystic fibrosis (CF) (Juan et al., 2005; Faure et al., 2018). This autosomal recessive disease, among other consequences, causes the secretion of altered respiratory mucus that works as a breeding ground for infections by *P. aeruginosa*, among other pathogens. These infections entail severe clinical and economic consequences and high mortality rates despite recent advances in CF treatments (Faure et al., 2018; Pallenberg et al., 2026). Hundreds of studies regarding adaptation, pathogenesis, clinical impact, and other implications of CF infection by *P. aeruginosa* have been published. Regardless, of particular interest for this review are those claiming that sex hormones influence the outcome of CF by affecting *P. aeruginosa* behavior (Chotirmall et al., 2012; Tyrrell and Harvey, 2020; Vidaillac et al., 2020; Al-Zawity et al., 2022), thus contributing to the so-called “CF gender gap” (Rosenfeld et al., 1997; Harness-Brumley et al., 2014). This phenomenon consists of worse outcomes for female patients, including a lower median life expectancy (Rosenfeld et al., 1997; Harness-Brumley et al., 2014). While this gap was initially attributed to physiological differences between women and men, recent studies demonstrate that it could also be due to variations in bacterial behavior depending on the sex of the patient. In this regard, it was demonstrated that female sex hormones estradiol and estriol readily induced the *in vitro* acquisition of mucoid phenotype by *P. aeruginosa* (featuring the hyperproduction of the exopolysaccharide alginate) through mutations in *mucA*. This outcome was attributed to estradiol exposure-driven impaired catalase activity and increased levels of hydrogen peroxide within *P. aeruginosa* cells, which in turn damage DNA, promoting mutations such as this one. The resulting mucoid phenotype renders *P. aeruginosa* more successful in the CF niche because of increased biofilm formation linked to alginate hyperproduction (Chotirmall et al., 2012). These phenomena were later confirmed using different CF-proceeding *P. aeruginosa* isolates, in which a concordant biofilm formation boosted upon estradiol exposure, was reported (Al-Zawity et al., 2022). Moreover, the levels of estradiol depending on phases of the menstrual cycle have been correlated with higher infective exacerbations and isolation of mucoid phenotypes in female CF patients, suggesting that oral contraceptives could be a therapy to reduce estradiol production and thus *P. aeruginosa* pathogenic success (Chotirmall et al., 2012). More recently, exposure to estrogens was shown to boost other *P. aeruginosa* virulence-related parameters, increasing the secretion of pyocyanin (phenazine with toxic effect on immune cells) by 80% and motility diameters more than 2-fold (Tyrrell and Harvey, 2020). Interestingly, estrogen receptor modulators such as tamoxifen were also shown to selectively inhibit *P. aeruginosa* motility, indicating that these drugs could be used as an adjuvant to curb estrogen-induced *P. aeruginosa* success for CF infection in women (Tyrrell and Harvey, 2020).

Vidaillac and colleagues contributed to the topic by demonstrating that, besides biofilm formation, motility, and rhamnolipid and elastase production *in vitro*, *P. aeruginosa* overall virulence is positively affected upon exposure to steroid sex hormones at physiological concentrations in a murine model of infection (Vidaillac et al., 2020). The proposed underlying mechanism was quite complex, involving the generation of membrane stress by the sex steroids themselves: the higher their polarity, the higher the envelopes’ disturbance, stress caused, and derived response. This situation was proposed to be sensed by surface signaling systems, including the *muc* operon, leading to the upregulation of genes involved in surface sensing (e.g. pili), quorum sensing, and biofilm formation (polysaccharides, alginate), among other virulence-related representatives. Polar sex steroids were also shown to alter membrane fluidity and composition, promoting lipid rafts formation and increased outer membrane vesicles release, which further boosted bacterial persistence and tissue damage (Vidaillac et al., 2020). Altogether, evidence about sex hormones improving *P. aeruginosa* pathogenic success for CF infection are probably among those most robust in this review. This is because they lean on clinical data of patients, murine models of infection, use of physiological concentrations of hormones and CF clinical strains, and complex assays to decipher the underlying molecular mechanisms.

Similarly, although with less robust levels of evidence (only *in vitro* assays) closely related molecules such as the corticosteroids fluticasone propionate and budesonide (anti-inflammatory drugs commonly administered to patients with CF or other pulmonary diseases) have been shown to boost biofilm formation in *P. aeruginosa* at concentrations attainable in treated patients (Jordana-Lluch et al., 2025). The proposed underlying mechanism was similar to that described by Vidaillac and colleagues, i.e. linked to alterations in membrane fluidity, activation of the *muc* operon and increased accumulation of the 2^nd^ messenger c-di-GMP (responsible for the transition from acute to chronic infection behavior) (Jordana-Lluch et al., 2025).

On the other hand, pre- and post-menopausal physiological concentrations of estradiol have been demonstrated to increase growth rate, biofilm formation, colonization and invasion of gingival epithelial cells (reaching values of almost 10–fold compared to controls) by the oral pathogen *Porphyromonas gingivalis* (Demirel et al., 2022). Interestingly, besides biofilm-related data, a common finding between the latter and the aforementioned investigation of Jordana-Lluch and colleagues (Jordana-Lluch et al., 2025), is that the studied steroids apparently reduced the intrinsic inflammatory properties of each starring species. This could be interpreted as a kind of double-edged sword, at least in the particular contexts of these studies: in the initial stages of infection, an attenuated inflammatory response could be advantageous for the pathogen because it would cause a lower immune activation, thereby enabling its initial silent growth and success for infection in the longer term (Demirel et al., 2022; Jordana-Lluch et al., 2025). However, these ideas should be confirmed through more robust evidence, i.e. the use of superior animal models.

Different studies approached the effects of sex hormones on the behavior of uropathogenic *Escherichia coli* (UPEC), providing interesting and varied results. In this sense, testosterone exposure (at physiological ranges, between 100 pg/mL and 60 ng/mL) significantly enhanced the virulence of UPEC in the *Caenorhabditis elegans* model, reducing survival of worms up to a third. This exposure also boosted growth, endotoxin release, biofilm formation, and colonization and invasion of bladder epithelial cells, consequently increasing the release of inflammatory mediators (Wu et al., 2024). Meanwhile, although an increased expression of adhesins and boosted colonization and invasion of bladder epithelial cells (up to ca. 5-fold compared to controls) was seen upon estradiol exposure of UPEC, a decreased elicited inflammatory response in these cells and attenuated virulence in *C. elegans* were reported (Engelsöy et al., 2021). Additionally, a general trend for increased expression of certain virulence related genes such as *usp* (uropathogenic-specific protein), *sfa/foc* (fimbrial adhesins), and *cnf1* (cytotoxic necrotizing factor 1) was also reported in UPEC upon estradiol exposure (Gümüş et al., 2019). Taking all these data together, one could think that the clearly increased virulence of UPEC when exposed to testosterone (Wu et al., 2024) should entail a more severe outcome for male patients and milder for women. However, if we consider the well-known gender gap in urinary infections (premenopausal women are up to 40 times more likely to get urinary infections than men in the same age range) (Deltourbe et al., 2022), one could think that the influence of sex hormones is not that simple, but rather the contrary. In this regard, it is known that after menopause and andropause (entailing an obvious decrease in estrogen and testosterone, respectively) the incidence of urinary infections significantly rises in both sexes, suggesting that, whatever the molecular bases are, both male and female sex hormones could break the success of UPEC for infection (Deltourbe et al., 2022). However, deciphering which part of these sex- and age-related outcomes is due to the specific effects of each hormone on the pathogen would be very complex. In this sense, since sex hormone levels influence many other features in the host (in addition to other obvious factors such as anatomy), pointing towards the bacterial virulence modulation as the unique cause of a better or worse urinary infection outcome would be overbold. Moreover, all the available results in this regard are difficult to reconcile, as they come from heterogeneous models such as invertebrate infection, epithelial cell assays, and immune cell studies, which likely reflect different aspects of bacterial pathogenesis. Therefore, the true net effect of sex hormones on UPEC virulence in humans remains unclear and worth to be delved into.

Continuing with the topic of sex-biased infections, a higher incidence of *Staphylococcus aureus* skin and soft tissue infections was reported in males (Castleman et al., 2018). In this regard, Luo and colleagues proposed through a murine model of skin infection, that the testosterone-mediated increase in *S. aureus* virulence could heavily contribute to this phenomenon (Luo et al., 2024). This increase was reflected in other parameters such as hemolytic activity, cytotoxicity, and oxidative stress resistance, and mediated by the upregulation of genes encoding α-hemolysin, phenol-soluble modulin, and accessory gene regulator quorum-sensing system components (*agrC, agrA, agrB, agrD*). Interestingly, deletion of *agrC* abolished the testosterone-induced increase in hemolysis and gene expression. Based on this fact and molecular docking simulations, authors suggested that AgrC protein is the *S. aureus* membrane receptor for testosterone (Luo et al., 2024). Therefore, despite this latter limitation (in silico instead of experimental data), the rest of evidence, even including a model with wildtype vs testosterone-deficient mice, seems robust enough to believe that this hormone acts as an enhancer of *S. aureus* success for skin infection, and thus as a promoter of worse clinical outputs.

### 2.2 Stress hormones and catecholamines

Stress hormones [cortisol, adrenaline (epinephrine), and noradrenaline (norepinephrine)] are chemical messengers released by the adrenal glands in response to physical or emotional threats, increasing heart rate, blood pressure and sugar levels, etc. Although dopamine is not a stress hormone but a neurotransmitter, it is chemically related to adrenaline and noradrenaline since they are all catecholamines. Therefore it is also approached in this section. Accordingly, studies dealing with catecholamine inotropes (drugs derived from natural catecholamines used for heart failure or shock) are also reviewed here. A remarkable review in the field of interplay between stress hormones and bacterial virulence was published by Boukerb and colleagues (Boukerb et al., 2021) and may be resourced for more detailed information. Thus, here we only review the most relevant and/or newer studies on the topic.

To start with, it has been known for decades that catecholamines boost the *in vitro* growth of several pathogens (*S. aureus, P. aeruginosa, E. coli, Klebsiella pneumoniae*, and *Yersinia enterocolitica*) (Lyte and Ernst, 1992; Belay and Sonnenfeld, 2002; Belay et al., 2003). Although the molecular bases for this phenomenon were not clear when these studies were published, they did represent some of the first evidence for host-proceeding mediators displaying activating effects on bacterial behavior. Turning to the topic in terms of species, *P. aeruginosa* is one of the pathogens showing a greater number of studies. Besides older data demonstrating a clear capacity of norepinephrine (at concentrations found in the gut, i.e. 50 μM) to increase PA14 strain growth, production of different virulence factors (pyocyanin, elastase), invasion of epithelial cells, and swimming motility involving the *las* quorum-sensing pathway (Hegde et al., 2009), some other interesting findings have recently been published. For instance, an increase in norepinephrine secretion due to extended contact lens use leading to the promotion of the pathogenesis of *P. aeruginosa* keratitis in a murine model was reported (Li et al., 2020). This worse output was attributed to norepinephrine-linked boosted adhesion and biofilm formation on the lens, but no underlying receptors/molecular mechanisms were investigated, limiting the impact of the results (Li et al., 2020). Meanwhile, Cambronel and colleagues demonstrated an enhanced virulence of the H103 strain towards *Galleria mellonella* larvae upon exposure to epinephrine (Cambronel et al., 2019). These phenomena could have more severe implications if we consider the conclusions of Monteagudo-Cascales et al., claiming that *P. aeruginosa* is chemotactically attracted to cathecholamines and serotonin, a phenomenon for which the TlpQ chemoreceptor was reliably demonstrated to be essential (Monteagudo-Cascales et al., 2025). Whether or not *P. aeruginosa* metabolizes host catecholamines into potent microbial chemo-attractants such as 3,4-dihydroxymandelic acid, as described for *E. coli* (Pasupuleti et al., 2017), remains to be investigated as the basis for the abovementioned chemotaxis phenomena (Monteagudo-Cascales et al., 2025). In accordance with all these studies, it was reported that antagonists of the adrenergic receptors (e.g. timolol), significantly decreased *P. aeruginosa* growth and biofilm formation as well as the expression of genes involved in the production of pyocyanin, *pslA* (essential for the biosynthesis of biofilm-related exopolysaccharides), and other virulence genes (*exoS*, *exoT*, *toxA*, and *aprA*) (Medina-Lopez et al., 2022). Obviously, the rationale for these findings is that these antagonists would also block the as yet unidentified *P. aeruginosa* adrenergic receptor. In this regard, some potential catecholamine receptor-like elements have been proposed in this species, such as the TonB-dependent iron transporters PirA and PiuA, among others (Perraud et al., 2022). This makes sense if we consider the catechol group of catecholamines, known for its high capacity to chelate iron, which would therefore be internalized through the aforementioned transporters. Thus, besides potential unidentified effects of the catecholamine itself, this parallel increased iron incorporation could support a boosted growth and virulence factor expression, given the essential role of this usually scarce metal for bacterial metabolism. However, these not mutually exclusive possibilities still need to be conclusively proven (Perraud et al., 2022). At any rate, all these evidence suggested interesting potentials for the exploitation of adrenergic receptor antagonists as a therapeutic strategy for chronically infected wounds (Medina-Lopez et al., 2022).

On the other hand, catecholamine inotropes were also shown to stimulate *P. aeruginosa* growth *in vitro*, producing up to 50-fold increases in bacterial counts (Freestone et al., 2012). This could be attributable to the sequestration of transferrin iron by the inotrope followed by its PirA/PiuA-mediated internalization (Perraud et al., 2022), and was associated to the hypeproduction of the siderophore pyoverdine (Freestone et al., 2012). Moreover, inotropes at clinically attainable concentrations were demonstrated to boost *P. aeruginosa* cytotoxicity and biofilm formation on endotracheal tubes, suggesting that these pharmaceuticals could be a potential contributory factor for ventilator-associated pneumonia (VAP) (Freestone et al., 2012) or catheter-related infections (Liu et al., 2022). These ideas, although logical, should be verified reliably to establish a clear cause-and-effect relationship between inotropes treatment and worse infection outcomes in the patient. Finally, Xiang et al. demonstrated the multiple stimulating effects of dopamine on *P. aeruginosa* pathogenic behavior: increased biofilm formation, motility, and boosted production of several factors such as alginate, rhamnolipids, proteases, and pyocyanin. However, the underlying mechanisms, beyond proposing dopamine as a potential exogenous quorum sensing-like signaling molecule, have not yet been identified (Xiang et al., 2024).

Finally, although numerous abovementioned studies demonstrated an increase in *P. aeruginosa* growth and virulence upon exposure to catecholamines, as can be seen in **Table 1** most of the evidence comes from *in vitro* or non-mammalian models, and often involves iron-mediated effects, raising doubts about whether catecholamines act as true signaling molecules and thus an specific receptor exists in this pathogen. Therefore, further research is needed to decipher whether or not an increased production of stress hormones by the patient actually aggravates the infections by *P. aeruginosa*.

Within *Enterobacteriaceae*, norepinephrine was proved to stimulate the production of Shiga-like toxins by *E. coli* O157:H7, and to boost growth rate and expression of the K99 pilus adhesin in enterotoxigenic *E. coli* (ETEC) (Lyte et al., 1996, 1997). It was later demonstrated that catecholamines were able to stimulate growth of commensal *E. coli* through host’s iron sequestration within the gut, which was proposed as a potential contributory phenomenon for trauma-induced sepsis (Freestone et al., 2002). Some years later, the QseC sensor kinase (making up a two-component system together with QseE) was identified in *E. coli* as the potential adrenergic receptor for epinephrine/norepinephrine, being lockable through the use of antagonists. Moreover, this receptor was found to be quite conserved in certain pathogenic species (e.g. *Shigella* sp., *Salmonella* sp., and *Haemophilus influenzae*) (Clarke et al., 2006). Nevertheless, these ideas were called into question by other studies, which suggested that QseC and QseE are not essential for *E. coli* and *Salmonella* to respond to stress hormones, and that they rather act as bacterial metabolism regulators (Pullinger et al., 2010; Hamed et al., 2022). On the other hand Ignatova and colleagues demonstrated an increase in biofilm production (up to two-fold compared to controls), adhesion, and motility linked to a metabolic activity of UPEC overstimulated upon norepinephrine exposure at concentrations found in the urine of healthy individuals (Ignatova et al., 2022). Similar results were later reported with different clinical ETEC isolates after incubations with norepinephrine and epinephrine (Niu et al., 2023).

Still within *Enterobacterales*, adrenaline was shown to modulate the global transcriptional profile of *Salmonella*, suggesting a significant impact on responses against antimicrobial peptides and oxidative stress (Karavolos et al., 2008). On the other hand, dopamine was demonstrated to work as a siderophore-like chelator increasing iron incorporation of *Salmonella* Typhimurium. The histidine sensor kinase QseC and transporters SitABCD were shown to be essential for this outcome, which promoted bacterial growth *in vitro* and virulence in a murine model of infection (bacterial counts from spleen and liver were increased at least 10-fold) (Dichtl et al., 2019). The use of clinically-relevant concentrations of dopamine, the data obtained with a superior animal model, and the reported abolition of effects when using KO mutants in the allegedly involved receptors/mediators, make of this study one of the most robust in this section (Dichtl et al., 2019).

Different studies demonstrate that adrenaline and noradrenaline are able to modulate the *P. gingivalis* behavior by stimulating the expression of genes related to iron acquisition (*hmuR*), oxidative stress resistance (*tpx, oxyR, dps, sodB*, and *aphC*), and other virulence factors (*hem, hagA*, and *ragA*) (Graziano et al., 2013). It was later shown that, although norepinephrine caused an increased expression of *P. gingivalis* virulence genes *in vitro*, this was not translated into increased deaths of larvae in the *G. mellonella* model, which suggests interpreting these results with caution (Moraes et al., 2022). Meanwhile, cortisol has been demonstrated to promote *P. gingivalis* proliferation, with the capture of toxic reactive oxygen species (ROS) by this hormone in initial growth stages being proposed as a potential underlying mechanism (Akcalı et al., 2014; Klomp et al., 2023). In the case of *Vibrio* genus, catecholamines displayed a significant growth stimulation capacity, although quite variable depending on the species (Nakano et al., 2006). Moreover, norepinephrine showed a significant power to stimulate the cytotoxic activity of *V. parahaemolyticus* (up to ca. 50% increase against Caco-2 cells) (Nakano et al., 2007). These effects could be attributed to the reported capacity of catecholamines to impact the *V. cholerae* proteome, mostly affecting proteins related to iron transport and homeostasis, energy metabolism, and signaling (Toulouse et al., 2019). Some studies have demonstrated impacts of stress hormones/catecholamines similar to those mentioned in this section, although in more isolated publications/species: boosting effects of norepinephrine on growth, adhesion to inert surfaces, and biofilm formation by *Acinetobacter baumannii* and *Aeromonas hydrophila*, including a fish infection model, and stimulation of expression of certain virulence-related parameters (e.g. protease activity) in the latter case (Inaba et al., 2016; Gao et al., 2019).

Returning to catecholamine inotropes, these drugs were shown to remove iron from transferrin to supply it into *S. epidermidis* cells (Neal et al., 2001), which was associated to an extraordinarily boosted capacity for adhesion to surfaces (increases up to thousands-fold in adherent cell counts) and biofilm formation (Lyte et al., 2003). Similar results, although in a milder fashion, were later obtained in *S. lugdunensis* challenged with dopamine (Frank and Patel, 2008). Interestingly, in the latter study, heparin was also shown to promote the detachment of *S. lugdunensis* and other *Staphylococcus* species cells from preformed biofilms, suggesting that certain combinations of intravenous pharmaceuticals could initially boost biofilm formation on catheters and later promote the systemic dissemination of these pathogens (Frank and Patel, 2008). These facts were shown to have wider implications, such as the capacity of these drugs to resuscitate staphylococci previously treated with rifampin or minocycline, a phenomenon reversible through exposure to catecholamine-receptor antagonists (Freestone et al., 2008).

On the other hand, norepinephrine at clinically attainable concentrations was shown to cause a significant stimulation of growth, biofilm formation, and virulence gene expression in *Streptococcus pneumoniae* (Sandrini et al., 2014). The pneumococcal iron uptake protein A (PiuA) was demonstrated to be a key factor in this process, linked to iron catchment by the catechol moiety of norepinephrine (Zhang et al., 2020). Although it is not a stress hormone or a catecholamine, the corticotropin-releasing hormone (CRH) is closely related to the former from a functional point of view, since it enables the release of cortisol by adrenal glands. Interestingly, this mediator improved pneumococcal pathogenic performance by stimulating growth, virulence in a murine model (more than 3 logs of increase in bacterial lung carriage), and expression of virulence-related parameters and factors such as capsule thickness (by 30-fold) and pneumolysin (Ndjom and Jones, 2015; Ngo Ndjom et al., 2017).

In turn, *Enterococcus faecalis* and *E. faecium* (including probiotic and pathogenic strains) have also been proved to be responsive to catecholamines, mostly in terms of increased biofilm formation and adhesion to Caco-2/TC7 intestinal cells and HaCaT keratinocytes, with certain variability depending on the isolates (Cambronel et al., 2020; Scardaci et al., 2022). A putative adrenergic sensor of catecholamines was proposed in *E. faecalis* (VicK protein), given its homology with the alleged *E. coli* adrenergic receptor QseC, and the molecular docking data obtained (Cambronel et al., 2020).

The impacts of catecholamines on the behavior of different pathogens belonging to *Actinobacteria* phylum (Barka et al., 2015) have also been approached. To start with, *Cutibacterium acnes* (formerly known as *Propionibacterium acnes*) has been studied regarding its behavior being modulated by epinephrine/norepinephrine. Interesting differences were obtained in this sense depending on the strain: while an acneic isolate was highly responsive to the presence of both catecholamines, showing significantly increased biofilm formation (between two- and three-fold compared to controls), a non-acneic strain experienced much milder changes. These data suggested that *C. acnes* could participate as an intermediary between stress mediators and acne appearance (Borrel et al., 2019). Meanwhile, although another typical human skin-colonizer, *Micrococcus luteus*, is not a typical pathogen, it can act as such in immunocompromised individuals showing responsiveness to stress hormones (Shi et al., 2023). More specifically, exposure to epinephrine at a physiological concentration increased 72-h biofilm biomass and cellular metabolic activity within the sessile community. This was related to complex changes in the transcriptome and in the protein, lipid, and polysaccharide profiles of the biofilm matrix, in which the amounts of polysaccharides and extracellular DNA were consistently increased (Gannesen et al., 2021, 2022). Finally, *Mycobacterium tuberculosis* has been demonstrated to respond to epinephrine with increased growth and formation of biofilm (ca. two-fold compared to control), which displayed differences in texture, composition, antibiotic resistance, and stress tolerance. Molecular docking analysis suggested that the extra-cytoplasmic domain of the MprB protein (belonging to the MprAB two-component system) could be the putative adrenergic sensor involved in these outputs (Lei et al., 2023), but this circumstance still needs to be experimentally demonstrated.

In conclusion to this section and as can be seen in **Table 1**, it should be noted that most studies used stress hormone/catecholamine concentrations well above physiological serum ranges. This has been justified because these hormones are highly unstable *in vitro*, making it necessary to use them at supra-physiological concentrations to yield a lasting effective dose during incubations. Furthermore, it has been suggested that micromolar ranges can be achieved in some microenvironments, and thus plasma concentrations would reflect spillover from tissues, underestimating those that may be actually affecting a bacterial population (Lyte, 2004; O'Donnell et al., 2006). In any case, given the scarcity of reliable data on the actual concentrations to which pathogens are exposed within the patient even in extreme situations (e.g. surgical stress, shock, treatment with inotropes), it cannot be assumed that they necessarily lead to worse outcomes of infection, in spite of all the clues published in this regard. Therefore, future studies to establish clearer cause-effect relationships between these endogenous mediators and increased pathogenic performance are needed.

### 2.3 Natriuretic peptides

Natriuretic peptides [e.g. Atrial Natriuretic Peptide (ANP), Brain Natriuretic Peptide (BNP), and C-type Natriuretic Peptide (CNP)] are hormones released by cardiomyocytes as defense mechanisms against heart failure, promoting vasodilation, diuresis, and natriuresis to reduce blood pressure. These peptides are known to readily modulate *P. aeruginosa* pathogenic behavior and, for instance, BNP and CNP were reported to increase glial cell necrosis after infection with this pathogen. This output was mediated by modifications in the levels of the signaling cyclic nucleotides cAMP and cGMP within *P. aeruginosa* cytosol, lipopolysaccharide (LPS) biosynthesis, and the essential participation of the cAMP-binding protein Vfr (Veron et al., 2007). Shortly after, these findings were reproduced to a large degree in *P. fluorescens* (Veron et al., 2008). Some differences were later reported regarding the effects caused on *P. aeruginosa* depending on the natriuretic peptide considered, which warrants caution in the interpretation of the following results. Whereas CNP increased its capacity to kill *C. elegans*, BNP did not affect this feature and, curiously, the production of pyocyanin was strongly inhibited by CNP (Blier et al., 2011). Moreover, both BNP and CNP significantly enhanced bacterial production of acylhomoserine lactone (AHL), suggesting a clear involvement of quorum sensing signaling in these phenomena. Participation of the regulator PtxR was demonstrated as a partial underlying base for these outputs as well (Blier et al., 2011). In contrast to most studies reviewed here, it was later demonstrated that exposure to BNP and CNP strongly decreased *P. aeruginosa* biofilm formation (to a ca. fifth part of the control) but without affecting bacterial growth. Isatin, an antagonist of human natriuretic peptide receptors, prevented this effect, for which the aliphatic amidase expression-regulating protein AmiC was proposed to be the *P. aeruginosa* orthologue involved in natriuretic peptide sensing (Rosay et al., 2015; Desriac et al., 2018).

Beyond *P. aeruginosa*, some recent studies approached the effects of natriuretic peptides on different skin colonizers/pathogens, mainly in the context of mixed-species biofilms. This poses a very interesting field approaching how human neuroendocrine systems can regulate the cutaneous microbial community, a topic of paramount importance to understand how the loss of homeostasis and derived dysbiosis can lead to infections. For instance, communities formed by *S. aureus* and *C. acnes*, or *S. epidermidis* and *C. acnes*, have been shown to be influenced by ANP and CNP, which caused an increased competitiveness of the latter species in both mixed biofilms (Gannesen et al., 2018; Ovcharova et al., 2021, 2023). Meanwhile, ANP has been shown to display disparate effects on monospecific and mixed biofilms of *S. aureus* and *Kytococcus schroeteri* (a typical commensal of the human skin microbiome that can act as an opportunistic pathogen). For instance, ANP boosted biofilm formation on hydrophilic surfaces like glass, whereas it dampened the formation of these communities on hydrophobic surfaces. Additionally, a combination of azithromycin and ANP led to an increase in cell aggregation in biofilms as well as matrix synthesis, but to a decrease in *S. aureus* proportion within the dual communities (Diuvenji et al., 2023). Likewise, BNP has been shown to influence different parameters of the human microbiome members *M. luteus* and *Alcaligenes faecalis* (Loiko et al., 2022): healthy levels of this natriuretic peptide (up to 250 pg/ml) were shown to increase bacterial tolerance to certain types of stress (e.g. H_2_O_2_, antibiotics, salinity, heat, and pH shock), whereas superior levels of ANP, which are secreted in situations of heart dysfunction, made bacteria less resistant (Loiko et al., 2022). By way of conclusion to this section, in contrast to sex and stress hormones, which tend to have a rather conserved array of effects boosting growth, virulence, and biofilm in most studies, natriuretic peptides display more variable and even contradictory impacts depending on the parameter, pathogen, and even conditions applied, warranting the need for more research.

### 2.4 Neurotransmitters

Neuropeptides are short amino acid chains produced by neurons that act as neurotransmitters and neuromodulators. They regulate vital functions such as pain, appetite, mood, stress, and memory. Key examples include oxytocin, endorphins, substance P, and neuropeptide Y. The direct antimicrobial activities of neuropeptides have been demonstrated against both Gram-negative and Gram-positive pathogens, with a detergent-like disruption of bacterial membranes as the main mediator mechanism. Neuropeptides also act as immunomodulatory factors and have been proposed to somehow educate our immune system to be tolerant against a myriad of species/situations, in order to enable the formation and maintenance of a healthy microbiome (Augustyniak et al., 2021). Neuropeptides dual and somehow paradoxical role acting as antimicrobial agents but also as enhancers of virulence in certain situations has recently been reviewed (Augustyniak et al., 2021). Thus we only highlight here the most relevant/newest studies in the field. For instance, the opportunistic pathogen *P. fluorescens* showed responsiveness to substance P, serotonin and epinephrine, although with milder outputs in the two latter cases (Biaggini et al., 2015). More specifically, swarming motility was slightly but significantly boosted upon exposure to substance P, whereas the release of IL-8 and the damages caused in the Caco-2/TC7 cell monolayer (in terms of permeability) were increased by 25% (Biaggini et al., 2015). Some other studies demonstrate that various neuropeptides such as substance P and calcitonin gene-related peptide (CGRP) condition staphylococcal behavior, which was proposed to have meaningful implications for skin homeostasis (N'Diaye et al., 2016a, b). CGRP stimulated certain *S. epidermidis* virulence features, being the threshold extremely low, below the mean concentrations of CGRP in blood (ca. 84 × 10^−12^ M) and skin (between 5.4 and 0.3 × 10^−12^ M). Moreover, changes in *S. epidermidis* behavior did not appear in parallel to a measurable change in virulence factor secretion but in surface properties (increased hydrophobicity), driving to boosted adherence to keratinocytes (by ca. 25% more than controls). Whereas cellular internalization and biofilm formation were reduced, the DnaK chaperone was experimentally identified as the *S. epidermidis* CGRP-binding receptor in this same study (N'Diaye et al., 2016a). In a parallel investigation substance P was shown to stimulate the virulence of *S. aureus* and *S. epidermidis* in a reconstructed human epidermis model, boosting adhesion to keratinocytes (N'Diaye et al., 2016b). Although the outcome was similar for both species, the underlying mechanisms were quite different: increased production of enterotoxin C2 was the likely key for *S. aureus* boosted virulence, whereas in *S. epidermidis*, the effect was seemingly substantiated mostly through increased biofilm formation. The thermo unstable ribosomal elongation factor Ef-Tu was reliably identified as the substance P receptor in both species (N'Diaye et al., 2016b). Finally, Mijouin et al. reported that substance P strongly stimulated cytotoxicity (more than 5-fold) of the member of the skin transient microflora and foodborne pathogen *Bacillus cereus*, and also its biofilm formation (ca. 33% more than controls). These results were associated with an increased release of collagenase and superoxide dismutase, and Ef-Tu was again experimentally demonstrated to be the substance P receptor (Mijouin et al., 2013).

Gamma-aminobutyric acid (GABA) is the primary inhibitory neurotransmitter in the human central nervous system, but it also showed virulence-modulating effects on *Pseudomonas* species. For instance, GABA at physiological concentration was reported to profusely increase *P. aeruginosa* cytotoxicity and killing capacity in the *C. elegans* model (more than two-fold) and boost its cyanogenesis through the over-expression of oxygen-scavenging proteins (Dagorn et al., 2013a). In a parallel study, GABA also affected the performance of different *P. fluorescens* strains, but not in exactly the same way as in *P. aeruginosa*: for instance, GABA increased the necrotic-like activity of a *P. fluorescens* strain on glial cells, but reduced its apoptotic effect and its biofilm formation on plastic. Besides, GABA apparently caused rearrangements in LPS structure, especially in lipid A of *P. fluorescens* (Dagorn et al., 2013b). Before these findings, a *P. fluorescens* periplasmic protein with high affinity for GABA and similarities with a subunit of mammalian GABA_A_ receptors had been identified as the likely receptor in this species (Guthrie and Nicholson-Guthrie, 1989; Guthrie et al., 2000). Meanwhile, PctC and McpG were later experimentally demonstrated to be the chemoreceptors mediating *P. aeruginosa* and *P. putida* chemotaxis towards GABA respectively, although without apparent effects on virulence (Rico-Jiménez et al., 2013; Reyes-Darias et al., 2015).

Dynorphins are a subtype of short opiate peptides that act as neurotransmitters and are robustly released during stress. A couple of studies demonstrated that, besides these effects, they can condition *P. aeruginosa* behavior to increase its infective success during stress situations in the host. Zaborina and co-workers demonstrated that dynorphin A (1–17) and some artificial derivatives boosted *P. aeruginosa* virulence through the activation of quorum sensing circuitry (Zaborina et al., 2007). This study also showed that exposure to the abovementioned dynorphin increased pyocianin production and virulence towards *C. elegans*, decreasing worm survival to a fourth part (Zaborina et al., 2007). The underlying pathway for these findings was mostly deciphered, involving the participation of the global transcriptional regulator MvfR and the quorum sensing-related quinolone signaling molecules *Pseudomonas* Quinolone Signal (PQS) or its immediate precursor 4-hydroxy-2-heptylquinoline (HHQ), for instance (Zaborina et al., 2007). Moreover, dynorphin (1−13) at concentrations attainable in gut during stress (∼0.4 μM) was shown to be the trigger for a defensive response similar to the one *P. aeruginosa* activates against antimicrobial peptides (Wright et al., 2017). This response consists of the up-regulation of efflux pumps and of proteins belonging to *pmrAB* and *arn* operons, involved in the modification of the structure of lipid A (LPS). The membrane receptor ParS from the two-component system ParRS was shown to be the initiator of these envelope defense mechanisms in response to dynorphin (Wright et al., 2017).

### 2.5 Cytokines

Given its fundamental role as a pro-inflammatory cytokine mostly related with the innate immune response, it is paradoxical that interleukin-1β (IL-1) shows pro-virulence effects on certain pathogens. For instance IL-1 boosted the growth of virulent but not avirulent *E. coli* strains (Porat et al., 1991), and slightly increased biofilm formation by the periodontal infection-causing *Aggregatibacter actinomycetemcomitans*. An IL-1β-binding surface-exposed lipoprotein, designated as bacterial interleukin receptor I (BilRI), was reliably proved to be the receptor responsible for this output in the latter species (Paino et al., 2011, 2013). Returning to *E. coli*, Engelsöy and colleagues demonstrated that UPEC behavior is readily modulated by the presence of IL-1β and also other inflammation/immune system cytokines such as TNF-α, IL-6, IL-8, and IFN-γ, although with surprising variations depending on the parameter analyzed: increased growth *in vitro* and virulence towards *C. elegans*, but reduced biofilm formation and hemolytic activity (Engelsöy et al., 2019).

IL-1β and a IL-1 receptor antagonist were also shown to boost *S. aureus* growth in both planktonic lifestyle (more than five-fold) and within biofilm (up to 2.5-fold) (Kanangat et al., 2001; McLaughlin and Hoogewerf, 2006). The growth-promoting regions of IL-1β were located in the amino acid residues 118–147 and 208-240 (Kanangat et al., 2001). Moreover, *S. aureus*, *P. aeruginosa* and *Acinetobacter* sp. were shown to increase their intracellular growth within human monocytes when these leukocytes were exposed to high concentrations of IL-1β or IL-6. This was interpreted by the authors as a possible mechanism to explain the frequent development of infections in individuals with intense and protracted inflammatory responses (Kanangat et al., 1999).

Again, despite its essential role in regulating immune responses and inflammation, IFN-γ was surprisingly shown to modulate the virulence-related behavior of *P. aeruginosa.* This phenomenon was specifically mediated by IFN-γ binding to the outer membrane porin OprF, which triggered the expression of the PA-I lectin (LecA). This element acts both as an adhesin and a toxin, facilitating both initial colonization and tissue damage (Wu et al., 2005). Moreover, IFN-γ challenge has been demonstrated to up-regulate the expression of putative virulence-related genes in *M. tuberculosis* (e.g. *vapC14* and *esxP*) and to increase its respiratory rate, secretion of extracellular vesicles, and growth in a granuloma model (Ahmed et al., 2022), as well as its survival within macrophages (Hop et al., 2024). Moreover, the so-called mycobacterial membrane protein large 10 (MmpL10) was robustly identified as the transmembrane binding partner of IFN-γ in this species (Ahmed et al., 2022; Hop et al., 2024).

Finally, the TNF-α cytokine, involved in the regulation of inflammatory processes, apoptosis, and immune response, was shown to alter some virulence properties of *Shigella flexneri* (the main cause of bacterial dysentery in developing countries) (Luo et al., 1993). Exposure to this cytokine caused enhanced invasion of HeLa cells and uptake by human and murine macrophages. However, these outputs were attributed to the interaction of TNF-α- *S. flexneri* cell complexes with TNF-α receptors present on eukaryotic cells rather than to exclusive effects of this cytokine on the bacterium (Luo et al., 1993). A similar phenomenon was later demonstrated for IL-8-*M. tuberculosis* complexes facilitating entry into human neutrophils (Krupa et al., 2015; Dziadek et al., 2016). TNF-α and IL-8 were also shown to affect *Neisseria meningitidis* performance by increasing its resistance to complement. Binding of these cytokines to two type IV pili subunits (PilE and PilQ) was demonstrated to be essential for this output (Mahdavi et al., 2013).

In conclusion to sections 2.3, 2.4, and 2.5, and as can be seen in **Table 2**, the studies on the starring endogenous mediators (natriuretic peptides, neurotransmitters, and cytokines) as enhancers of bacterial pathogenic success seem to be slightly more robust than those reported for stress hormones/catecholamines. Thus, although superior animal models remain scarce, the widespread use of physiological concentrations and clinical isolates, and the numerous reliable assays regarding receptors recognizing the endogenous chemicals and enabling the reported effects (immunoprecipitation, mass spectrometry, knockout mutants, calorimetric titration, etc.) suggest that the findings in this field could have real relevance. In any case, it would be of great interest to promote research on the topic in order to establish a clear cause-and-effect relationship between the presence of certain concentrations of the starring endogenous mediators (found in situations of stress, heart failure, etc.) and worse clinical outcomes. The use of superior animal models and analysis of clinical data from real patients should be addressed in this regard.

## 3 Synthetic chemicals

In this section, the most relevant phenomena of synthetic substances incorporated into the human body in significant doses on more than a few occasions and displaying notorious bacterial virulence-boosting effects are reviewed. These chemicals –some of which display intrinsic nocive effects on our health– can be found in tissues because of voluntary ingestion/inhalation (e.g. the consumption of pharmaceutical products or smoking), but also due to involuntary exposure, as happens with volatile chemicals from routinely used products or even pollutants/additives present in food (Ilbäck and Friman, 2007; Lavezzi and Ramos-Molina, 2023; Shetty et al., 2023). An overview of the most relevant published data regarding the effects of these chemicals on bacterial pathogenic behavior is shown in **Table 3**. As will be seen in this table and the following sections, although in comparison with previous chemical mediators the use of superior animal models is more widespread, the investigation into the molecular bases of virulence modulation phenomena has been quite superficial (usually involving only transcriptomic analysis), leaving gaps in knowledge that must be filled.

### 3.1 Endocrine disruptors

Artificial endocrine disruptors are chemicals used in several human products (e.g. additives of polyvinyl chloride plastics; cosmetic stabilizers; composition of certain pesticides, detergents, flame retardants, paints, food packaging, etc.). They have been described to interfere with the proper functioning of hormones, leading to adverse health effects including reproductive, developmental, and metabolic disorders (Kovacs et al., 2025). Some typical examples are bisphenols, parabens, phthalates, and triclosan, and besides their presence in many elements in our daily routine, they are also widespread in the environment as pollutants, making it difficult to quantify the exposure to them. Besides their intrinsic health-related effects, recent evidence suggests that they could have additional impacts, in this case boosting bacterial pathogenic power (Thiroux et al., 2023a).

Since phthalates are one of the predominant endocrine disruptors present in humanized environments, the study of its effects on bacterial pathogens has been profusely approached. For instance, a dose-dependent increased biofilm formation by *P. aeruginosa* (up to two-fold compared to controls) has been reported upon exposure to different phthalates and phthalate substitutes (considered less toxic because of their limited volatility) (Louis et al., 2022). In this same study, some of these chemicals were shown to enhance *P. aeruginosa* membrane fluidity and alter cell morphology, albeit underlying mechanisms were not investigated (Louis et al., 2022). Moreover, in a coeval study, some phthalates were shown to upregulate (up to ca. twenty-fold) the expression of genes encoding for quorum sensing elements, extracellular polymer secretion, and oxidative stress resistance, which was translated into significantly boosted biofilm densification and resistance to chlorine (Wang et al., 2022a).

Phthalates have also been studied regarding their potential effects on the biology of another respiratory pathogen as *Legionella pneumophila*, the causative agent of Legionnaires’ disease (Crépin et al., 2023). At certain concentrations, some of the chemicals tested, such as acetyl tributyl citrate and Di-n-butyl phthalate, increased motility and the minimum inhibitory concentration (up to two-fold) of some antibiotics such as rifampin or levofloxacin (Crépin et al., 2023). In any case, the first species in which phthalates were shown to have virulence-regulating effects was *Helicobacter pylori*, the causative agent of chronic infections of the stomach/duodenal lining (Lin et al., 2013). Exposure to Di(2-ethylhexyl) phthalate increased the cytotoxicity of this pathogen against gastric epithelial cells, reducing survival ca. two-fold compared to the toxic effects of the chemical alone, and ca. four-fold compared to infection without phthalate exposure (Lin et al., 2013).

Returning to *P. aeruginosa*, other endocrine disruptors [bisphenol A, dibutyl phthalate, ethylparaben, methylparaben and, paradoxically, triclosan (used as an antimicrobial agent in personal care, textile, and plastic products)] at concentrations found in water resources as pollutants have been tested, displaying some striking effects (Thiroux et al., 2023b): swimming and swarming motilities decreased ca. 75% upon exposure to ethylparaben and dibutyl phthalate, respectively, but other parameters showed significant increases, such as two-fold greater adhesion to lung cells upon exposure to ethylparaben, or increased biofilm formation (up to ca. two-fold) upon exposure to bisphenol A, dibutyl phthalate, and ethylparaben. These outputs were interpreted as a phenomenon of simultaneous decrease of mobility enabling a consequent increase in cell adhesion and biofilm formation, which may promote establishment of the pathogen in individuals exposed to relevant doses of these endocrine disruptors (Thiroux et al., 2023b).

On the other hand, prolonged exposure (up to 26 days) of *Acinetobacter calcoaceticus* and *Stenotrophomonas maltophilia* to methylparaben, propylparaben, and butylparaben has been demonstrated to increase cellular culturability, density, and thickness of biofilms by more than 200% (Pereira et al., 2023). Moreover, swimming motility and production of protease and gelatinase by *S. maltophilia* were boosted up to 141%, 41%, and 73%, respectively, when exposed to methylparaben (Pereira et al., 2023). Regarding the main causative agent of dental caries, *Streptococcus mutans*, bisphenol A glycidyl methacrylate improved its adhesion capacity and glucan synthesis, which could contribute to biofilm formation (Kim et al., 2019), in a similar way to what had been previously demonstrated for subinhibitory triclosan on this species and *S. aureus* (Bedran et al., 2014; Syed et al., 2014). The *S. mutans*-related outputs were associated to a significant up-regulation of genes related to adherence and biofilm formation, such as *comD, gtfC*, and *luxS* (Bedran et al., 2014). In the case of *S. aureus* authors concluded through a rat model that triclosan exposure could promote nasal colonization in patients (Syed et al., 2014).

### 3.2 Opioids

Opioids are powerful painkillers derived from the poppy or synthetically produced, whose typical examples are morphine, oxycodone, fentanyl, and codeine. Morphine was demonstrated to act as a potent booster of *P. aeruginosa* virulence in a murine model of gut-derived sepsis: whereas controls caused no mortality at 72h, morphine-treated infected mice were all dead at 48h, for which *P. aeruginosa* seemed to develop improved mucus production-suppressing and gut epithelial barrier-disrupting effects. Interestingly, morphine also exerted a chemotactic effect towards *P. aeruginosa* (Babrowski et al., 2012). Similar results were obtained with the k-opioid U-50488, which caused a significant increase in pyoverdin and pyocianin production and decreased survival of *C. elegans* infected by *P. aeruginosa* PAO1. These facts were linked to an increased expression of the *pqsABCDE* operon (and thus boosted production of the quorum sensing signal PQS), and of other virulence-related genes (Zaborin et al., 2012). Finally, morphine was also shown to boost both *in vitro E. faecalis* adhesiveness and collagenase production (more than five-fold), which was translated into impaired anastomotic healing, gross leaks, and significantly worse outcomes in a rat model (Shakhsheer et al., 2016).

### 3.3 Benzodiazepines

It is known that sedation with benzodiazepines has eventual side-effects on the host (e.g. immunity alterations and nervous/mechanical responses) that increase the risk of VAP (Barceló et al., 2025). However, it has recently been shown that these sedative drugs also display modulating effects on *P. aeruginosa* behavior that could contribute to its success for VAP development. Upon exposure to clinically-attainable concentrations of diazepam or midazolam, significant increases in biofilm formation (up to two-fold) on plastic plates and endotracheal tubes were seen *in vitro* (Barceló et al., 2025). These outputs were supported by the upregulation of biofilm-related genes/KEGG pathways and increased c-di-GMP accumulation, suggesting the BZ-dependent boosted formation of biofilm reservoirs which could potentially increase bacterial release to low airways and thus VAP progression (Barceló et al., 2025). However, a *P. aeruginosa* receptor for benzodiazepines has never been identified, unlike in *P. fluorescens*. In this regard, the mammalians’ translocator protein (TSPO), designated as a peripheral-type benzodiazepine receptor, is a protein mainly located in the outer mitochondrial membrane of eukaryotic cells. Interestingly, Chapalain et al. demonstrated the existence of a *P. fluorescens* TSPO orthologue that shares common structural and functional characteristics with its mammalian counterpart. As such, it could be the key to the increased adhesion of this species to glass/eukaryotic cells and biofilm formation reported upon stimulation with PK 11195, an artificial ligand of mitochondrial TSPO (Chapalain et al., 2009). In this same study, the authors reported that bacterial TSPO seems to be widely distributed among other human pathogens such as *Bacillus anthracis*, *Clostridium perfringens*, *L. pneumophila*, and *S. haemolyticus* (Chapalain et al., 2009). Although no data regarding its specific responsiveness to benzodiazepines have been published, TspO from *B. cereus* has been suggested to play important roles in the regulation of its pathogenic performance (Duport and Armengaud, 2025).

### 3.4 Cigarette-related chemicals

Traditional cigarette smoke and e-cigarette vapor in general, but their starring compound/additive in particular (nicotine), have been profusely studied in terms of their capabilities to boost bacterial virulence. Thus there is a great body of evidence demonstrating that harmful effects of smoking are extensible to host-pathogen interaction (e.g. alterations in respiratory epithelia and immunity finally favoring infection) (Bagaitkar et al., 2008) but also to the pathogen’s biology itself, entailing greater risks and worse outputs in smoker individuals, as will be reviewed here.

On the one hand, tobacco smoke exposure was shown to boost biofilm formation *in vitro* in *P. aeruginosa* PAO1 strain (ca. 50% more than controls), provided that this challenge was repetitive (Goldstein-Daruech et al., 2011). This output was later related to a significantly increased expression of genes intimately linked to biofilm formation such as *pilF*, *flgK*, *algC*, and *lasI* (Antunes et al., 2012). Moreover, cigarette smoke extract was also shown to enhance *P. aeruginosa* virulence and biofilm mass (ca. three-fold), although paradoxically, it also slowed bacterial growth (Chien et al., 2020). In this same study it was reported that while none of the mice infected with *P. aeruginosa* exposed to smoke extract were alive at day six, ca. a quarter of controls survived in the pneumonia model. Moreover, the reported hyper-expression of the *tpx* gene (essential for defense against oxidative stress) after exposure to smoke was related to a greater resistance to neutrophil killing *in vitro*, since oxidative burst is one of the microbicide mechanisms used by these leukocytes (Chien et al., 2020).

The effects of smoke and nicotine have also been studied in the context of oral pathogens such as *P. gingivalis*, as expected due to the well-known phenomenon of a great prevalence of mouth pathologies in smokers (Beklen et al., 2022). For instance, cigarette smoke was shown to cause a significantly increased translation of the *P. gingivalis* major fimbrial antigen (FimA protein) and to suppress the production of capsular polysaccharides, which posed optimal facts for biofilm formation (Bagaitkar et al., 2010). Moreover, FimA was shown to cause little inflammation when recognized by TLR2 and, what is more, FimA recognition abrogated the pro-inflammatory response after stimulation by other TLR-2-specific agonists. Therefore, the authors concluded that cigarette smoke clearly promoted *P. gingivalis* colonization and infection, outcomes for which an impaired inflammatory response was likely an important contributory factor (Bagaitkar et al., 2010). In accordance, it was later demonstrated that tobacco smoke also promoted the dual species *Porphyromonas gingivalis-Streptococcus gordonii* (a key commensal in dental plaque) biofilm formation in a FimA-dependent manner, exhibiting a lower pro-inflammatory power than control biofilms (Bagaitkar et al., 2011). Nicotine, specifically, was later demonstrated to modulate the *P. gingivalis* proteome, causing a particular increase in production of mediators involved in virulence, metabolism, oxidative stress, and protein synthesis and folding, which likely contributed to the aforementioned outputs (Cogo et al., 2012). Finally, the effects of nicotine on *Lactobacillus casei* (species related to progression of existing caries especially in dentin, since it thrives in acidic environments produced by other bacteria) and other cariogenic/periodontal pathogens were analyzed by DuBois et al. These authors demonstrated an increased biofilm formation (up to ca. five-fold) in *L. casei, E. faecalis, Actinomyces viscosus*, and *Rothia dentocariosa*, and boosted planktonic growth in the latter three species, upon nicotine exposure (DuBois et al., 2014). Still related to oral pathogens, cigarette smoke and carbon dioxide-rich environments have long been known to significantly increase the growth of *S. mutans* and *S. sanguis* in terms of colony diameter (ca. 60% more compared to control), with greater effects reported for high nicotine content brands (Zonuz et al., 2008). Accordingly, tobacco smoke concentrate was demonstrated to promote adhesion (3.37–fold) and biofilm formation (1.60-fold) by *S. mutans* on orthodontic brackets (Baboni et al., 2010), and even more pronounced results were obtained after e-cigarette vapor exposure (Rouabhia and Semlali, 2021). Nicotine in particular showed similar effects on *S. mutans*, and, although a concentration of 8 mg/ml exhibited a certain repressor capacity on planktonic growth, it increased biofilm thickness (up to two-fold) and metabolic activity (up to six-fold) (Huang et al., 2012). These booster effects of nicotine on *S. mutans* biofilm production were later corroborated in dual species communities with *S. sanguinis* (Li et al., 2014). Nicotine also positively affected growth, biofilm formation and cell aggregation of the non-pathogenic species *S. gordonii*, believed to initiate dental plaque and thus provide binding sites for later pathogen colonizers such as *S. mutans* (Huang et al., 2014). In accordance, increased production of extracellular polysaccharides and higher cell counts from biofilms formed by *S. mutans* upon exposure to nicotine were later reported (Huang et al., 2015). Finally, nicotine was also demonstrated to increase the expression of virulence-related *ldh* and phosphotransferase system (PTS)-associated genes as well as lactate production by *S. mutans*, which were interpreted as triggers for increased dental caries in smoking individuals (Li et al., 2016).

Still within the *Streptococcus* genus but beyond oral pathologies, Mutepe and colleagues demonstrated that tobacco smoke boosted *S. pneumoniae* biofilm formation by 50% and also caused a substantial attenuation of the pore-forming interactions of its toxin pneumolysin. This was interpreted as an advantage for initial colonization, as it would elicit a milder inflammatory and alarm-immune response (Mutepe et al., 2013). These phenomena were dissected from the underlying basis perspective and, for instance, the participation of the two-component regulatory system 11 was demonstrated (Cockeran et al., 2014). Moreover, a kind of survival response including the hyper-expression of genes encoding for detoxification enzymes, stress resistance proteins, osmoregulator transporters, and efflux pumps was also reported upon exposure of *S. pneumoniae* to tobacco smoke (Manna et al., 2018; Cockeran et al., 2020). Interestingly, exposure of this pathogen to e-cigarrete vapor (with vs without nicotine) had some particularities in that, for instance, no changes in virulence were seen for nicotine-containing vapor beyond boosted biofilm formation (≈50% increase compared to controls). However, transcriptomics displayed nicotine-dependent alterations, mostly in genes encoding for heat shock proteins, oxidative stress response proteins, and two-component systems (Bagale et al., 2020). Similarly, no alterations in *E. coli* overall virulence after exposure to tobacco smoke were reported using a *Drosophila* infection model, although different alterations in its transcriptome, mostly affecting two-component systems, were found (Shiratsuchi et al., 2024).

*Staphylococcus aureus* also displays clear responsiveness to smoke, nicotine, and vapor challenges. For instance, *S. aureus* biofilm formation was shown to be boosted upon cigarette smoke (up to ca. four-fold, with variations depending on the strain), mostly via oxidative stress-related responses (Kulkarni et al., 2012). More specifically, a decreased expression of the quorum-sensing *agr* system, involved in biofilm dispersal, was reported as well as an upregulation of biofilm inducers such as *sarA* and *rbf*. In parallel, a transcriptional induction of bacterial oxidoreductases devoted to counteract the toxic effects of ROS present in smoke was also seen. Up-regulation of the *fnbA* gene (encoding fibronectin binding protein A) was also outlined as the basis for the boosted binding of *S. aureus* to immobilized fibronectin and human lung cells reported upon smoke exposure (Kulkarni et al., 2012). Moreover, although cigarette smoke had some growth-inhibiting effects on methycillin-resistant *S. aureus* (MRSA), it also induced more adherent, invasive, immune-resistant phenotypes (increased resistance to macrophage and neutrophil killing and to the effects of antimicrobial peptides).These facts were translated into more severe outcomes in murine models of pneumonia (e.g. four-fold more mortality than controls). Interestingly, very similar outputs were obtained in a parallel study analyzing the exposure of *S. aureus* to e-cigarette vapor (McEachern et al., 2015; Kulkarni et al., 2016; Hwang et al., 2016). Several virulence genes related to these phenomena were found to be significantly up-regulated upon exposure to tobacco smoke/e-cigarette vapor, such as those encoding for adhesins (clumping factor B and fibronectin- and fibrinogen-binding proteins A and B), staphylococcal protein A, staphylocoagulase, and nuclease (Kulkarni et al., 2016; Hwang et al., 2016). Although these facts suggest a boosted acute virulence profile for *S. aureus* exposed to cigarette smoke, other recent data support the idea of slightly attenuated smoke-induced virulence performance enabling persistent infection (Lacoma et al., 2019): although exposure of *S. aureus* to smoke was associated with augmented biofilm formation, invasion, and persistence within A549 bronchial alveolar epithelial cells, production of toxins was significantly downregulated (Lacoma et al., 2019). Nicotine in particular was also studied regarding its effects on the behavior of *S. aureus*, and results were partially in accordance with those using whole cigarette smoke, although with some curious nuances: a dose-dependent increase in attachment, extracellular DNA release, and biofilm formation was seen in parallel to an attenuated virulence profile (impaired invasion of A549 cells and decreased expression of several virulence factor-encoding genes), and a higher rate of autolysis entailing a greater percentage of dead cells within biofilms (Shi et al., 2019). These results, which are difficult to interpret, clearly demonstrate the complexity of how the virulence and pathogenic success of bacteria are modulated in the presence of smoke/vapor. This suggests that conclusions in this regard should be drawn cautiously and highlights the need for further investigation.

Moving to another meaningful pathogen such as *M. tuberculosis*, exposure to nicotine has also been associated with an increased expression of genes linked to resistance against antimicrobial peptides and other virulence-related ones. A booster effect for both extracelular and intracellular growth of this pathogen was also reported for nicotine in a dose-dependent manner, phenomena that could jointly contribute to the increased risk of smokers developing tuberculosis (de Haro-Acosta et al., 2021; Rivas-Santiago et al., 2023).

Finally, some studies analyzed the effects of smoke/vapor not on a single pathogen but on a collection of relevant species with comparative purposes. For instance, Goldstein-Daruech and colleagues demonstrated that clinical isolates of different pathogens (*P. aeruginosa, S. aureus, K. pneumoniae, K. oxytoca, S. pneumoniae, Serratia marcescens*, and *Proteus mirabilis*) from chronic rhinosinusitis patients produced biofilm *in vitro* to a greater extent proportional to two factors: the patient being a smoker, and the length of exposure to smoke *in vitro* (Goldstein-Daruech et al., 2011). Meanwhile, Gilpin and co-workers studied the effects of e-cigarette vapor and cigarette smoke on the virulence of relevant respiratory pathogens (*H. influenzae, S. pneumoniae, S. aureus*, and *P. aeruginosa*), demonstrating significant increases on biofilm formation and mortality of *G. mellonella* larvae upon exposure to both stimuli (Gilpin et al., 2019).

In conclusion to this section and regarding the bacterial pathways underlying all the reviewed outcomes, although no specific receptors for nicotine have been identified as yet, the effects of smoke/vapor seem to be intimately related to the oxidative stress entailed. Bacteria sense the high presence of oxidants within these volatile stimuli, and respond to them by activating pathways that confer resistance to oxidative stress. This activation also makes them more resistant to the host’s immune system, for instance to the oxidative burst used by neutrophils to kill bacteria. Additionally, exposure to oxidant mutagens, such as those present in cigarette smoke, can damage bacterial DNA, triggering the activation of the SOS response DNA repair system, which in turn may favor selection for adaptive changes driving, for instance, towards boosted biofilm formation. Moreover, evidence suggests that some two-component systems play a key role in the process, through membrane receptor histidine kinases and transcriptional regulators that sense and respond to environmental changes through modulation of expression of several genes under the control of the latter (Kulkarni et al., 2012; Cockeran et al., 2014; Lacoma et al., 2019; Bagale et al., 2020; Chien et al., 2020; Shiratsuchi et al., 2024). At any rate, more research is needed to understand which specific compounds within e-cigarette vapor exposure could contribute to the modulation of bacterial behavior in addition to the high presence of ROS, and through which underlying mechanisms, as well as the exact pathways involved in the impact of nicotine on bacterial performance. Gaining knowledge into these topics could provide clues useful to design novel therapeutic strategies to curb the increased pathogenic success upon exposure of bacteria to e-cigarettes/tobacco.

## 4 Other compounds with virulence-boosting capacity

In this last section, we gather isolated studies evidencing that certain mediators not included in the previous sections are able to boost bacterial pathogenic success as well. For instance, in contrast to denatured insulin, Humulin^®^ (commercial insulin for diabetes treatment) and purified insulin were demonstrated to increase *P. aeruginosa* biofilm formation *in vitro*. This phenomenon, together with the described insulin treatment-driven modulation of the immune response, could contribute to perturb wound infection resolution in diabetic patients (Watters et al., 2013, 2014). Meanwhile, non-transferrin-bound iron (often found in long term-stored donor blood, because of progressive erythrocyte lesion) was shown to enhance *P. aeruginosa* proliferation and biofilm formation *in vitro*, as well as mortality in catheterized mice transfused with long term-stored blood. These phenomena were related to the well-known essential role that iron bioavailability plays to enable full virulence and biofilm formation in several pathogens (La Carpia et al., 2022; Cassat and Skaar, 2013). Besides, adenosine (a typical end-product of intestinal hypoxia that is released by host cells in situations such as surgical stress) was found to boost the expression of a well-known virulence factor in *P. aeruginosa*, PA-I lectin/adhesin (*lecA*) (Kohler et al., 2005; Patel et al., 2007). Interestingly, exposure of *P. aeruginosa* to individual structural tissue components demonstrated that fascia specifically boosted the production of virulence-related mediators such as pyocyanin and the siderophore pyochelin (Kim et al., 2015).

In turn, the human hormone gastrin at physiological concentrations was shown to work as a specific growth factor for *H. pylori*, clearly suggesting this phenomenon as a key factor for the adaptation of this pathogen to its unique habitat, although the specific bacterial receptor was not identified (Chowers et al., 1999). Interestingly, regarding another gastrointestinal pathogen such as *S. enterica*, exposure to salad leaf juices was reported to boost its motility, biofilm formation, and attachment to leaves by >220%, 250%, and >350%, respectively. These data suggest that *S. enterica* gains a key growth advantage from fluids released by salad leaf damage (although the specific molecules responsible were not identified), obviously increasing risks of infection for consumers (Koukkidis et al., 2016).

On the other hand, clinically-relevant concentrations of paracetamol enhanced biofilm formation in several clinical strains of *S. aureus* (particularly among those belonging to the epidemiologically relevant clonal complex 8). This output was attributed to the significant rise in N-acetyl-glucosamine-containing components of the biofilm matrix (Sultan et al., 2021). Later, exposure to several commonly used drugs was shown to increase biofilm formation in extended-spectrum β-lactamase-producing *S. aureus* isolates on different surfaces. Although variations were meaningful depending on the strain, surface, and concentration used, the drugs that provided more pronounced increases (ca. two-fold) were the hypocholesterolemic lovastatin, the antihistamine loratadine, the anti-inflammatory diclofenac, and the antihypertensive verapamil (Carmona-Orozco and Echeverri, 2024). Moreover, exposure to per- and poly-fluoroalkyl substances (PFAS) [important environmental contaminants with bio-accumulative features (half-lives > five years in serum of exposed individuals)] was shown to boost *S. aureus* virulence. This was reflected in reduced survival rates in a *C. elegans* model, an outcome linked to hyper-expression of the virulence gene regulator *saeR* and *hla* (encoding for α-hemolysin) (Mangu et al., 2022).

Finally, epidermal growth factor (EGF), a potent mitogen for a variety of eukaryotic cells, was shown to boost growth of *M. avium* and *M. tuberculosis in vitro* and within macrophages. A 35 kDa EGF-binding protein with significant similarity to streptococcal glyceraldehyde-3-phosphate dehydrogenase (GAPDH) was reliably identified in these species as the receptor (Bermudez et al., 1996). *M. tuberculosis* GAPDH was later shown to also function as a receptor for human lactoferrin, posing a clever strategy for the capture and internalization of host iron and probably to benefit from it in order to boost virulence (Malhotra et al., 2017).

## 5 Discussion and concluding remarks

**Figure 2:**
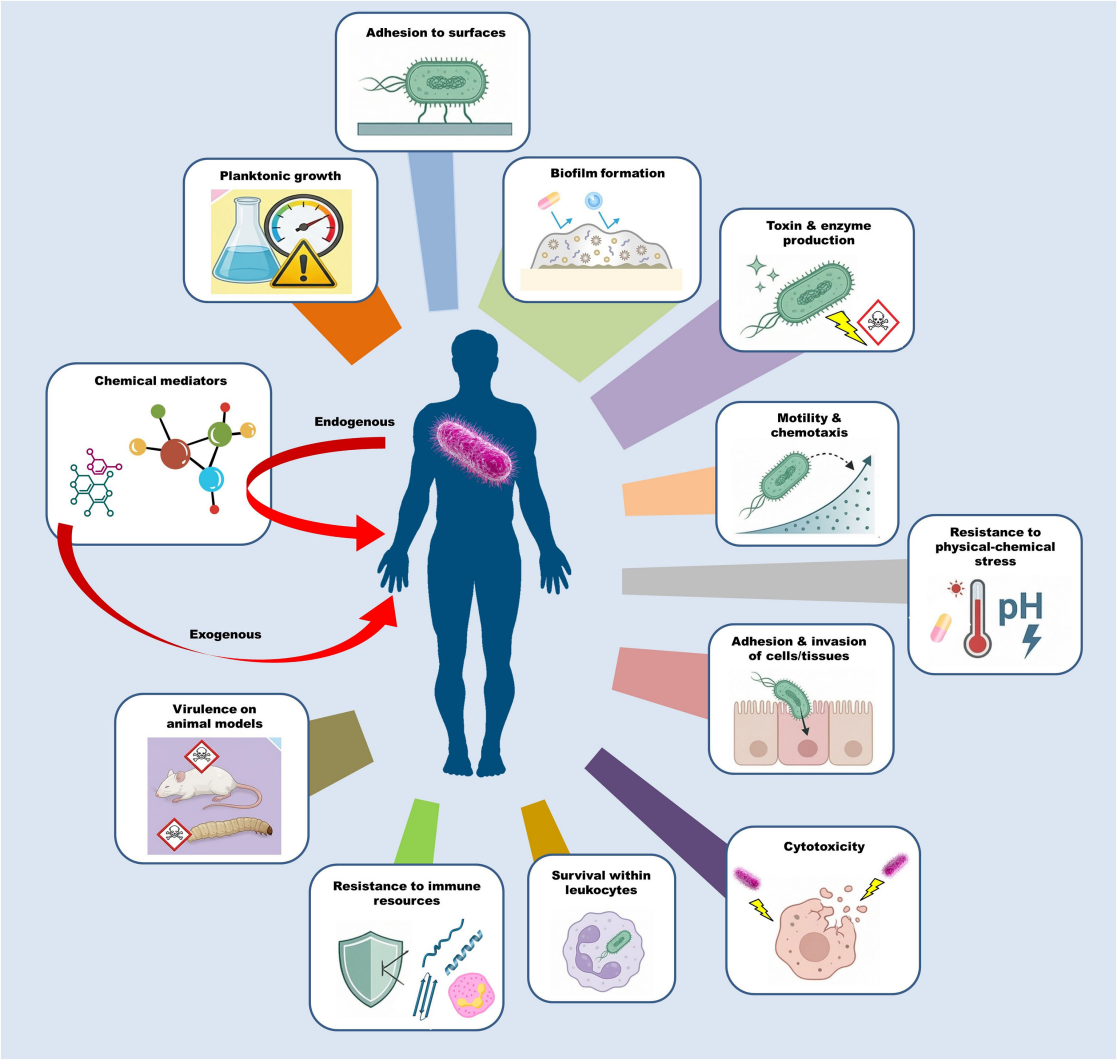
Overview of the main phenomena of bacterial pathogenic power improvements upon exposure to endogenous/exogenous chemicals reviewed here. Width of the coloured parallelograms under the virulence-related parameters is proportional to the number of studies demonstrating a significant increase in each one.

Data integrated throughout this review make it evident that several natural/synthetic chemicals potentially found at relevant concentrations within the human body display significant pathogenic-boosting effects on clinically meaningful bacterial species. These effects are reflected in the multiple phenomena of increased values of virulence-related parameters reviewed here, an overview of which is displayed in **Figure 2**. Regardless of the pathogens analyzed, the most usual effect in the studies reviewed is an increase in biofilm-related parameters, with ca. 50 different papers demonstrating data in this regard, followed by results of boosted release of toxins/enzymes, planktonic growth, virulence in animal models, and adhesion/invasion of cells (ca. 20 studies each). In any event, as can be seen in **Tables 1**-**3**, since these virulence-modulating impacts have been analyzed in many cases only through *in vitro* assays and/or invertebrate animal models, low numbers of bacterial strains, and in silico simulations for the potential receptors, it is difficult to reliably quantify their biological relevance from the clinical perspective. Thus, the possibility that alterations in some virulence parameters under the used experimental conditions could not necessarily lead to a worse clinical outcome in real patients must be considered. Therefore, conclusions from some of the reviewed studies should be taken with caution, since neither superior animal models nor clinical data proceeding from real patients have been used, making difficult to draw clear cause-effect relationships between presence of certain mediators and booster impacts for pathogenensis. These circumstances pose the knowledge gaps and limitations for the available data that research should solve in a near future. Another critical point, mostly applicable to the synthetic chemicals we incorporate as ingested/inhaled pollutants, is that it is difficult to find data concerning the real concentrations they reach within our tissues and thus to estimate whether or not contact between them and bacteria is sustained for a long enough time to exert significant virulence-modulating effects. Additionally, the use of supra-physiological concentrations of endogenous mediators (especially in the case of stress hormones and catecholamines) in most studies, makes raise some reasonable doubts about the actual applicability of the data obtained (**Table 1**). Thus, expanding our knowledge in these fields is necessary in order to decipher the real dimension of the threat likely posed by the phenomena reviewed here. In any case, despite the aforementioned limitations of certain studies, it is clear that the reviewed data provide clear clues about the potentials that many chemical mediators could display to enhance bacterial pathogenic success. Consequently, as a precautionary measure until we have more robust experimental data, virulence-enhancing effects linked to the endogenous/exogenous chemicals gathered in this review should be considered as potential risk factors for certain infections/patients.

On the other hand, although their analysis was beyond the objectives of this review, similar phenomena have been reported for different human origin chemicals acting as enhancers for antibiotic resistance, both from the point of view of the derived phenotype and also of the horizontal transfer of resistance determinants (Cohen et al., 1993; Tavío et al., 2004; Peterson et al., 2011; Tavío et al., 2012; Inaba et al., 2016; Ambrose et al., 2018; Henly et al., 2019; Wang et al., 2022b; Shi et al., 2022; Yin et al., 2023; Du et al., 2025; Chen et al., 2025). These obviously pose additional factors that may aggravate the consequences of infections and thus be worthy to be put under surveillance, together with the virulence-increasing phenomena reviewed.

Much information is still missing concerning the bacterial receptors/pathways/mechanisms enabling many of the virulence-boosting phenomena reviewed here. This fact is especially striking in the case of endocrine disruptors and certain pharmaceuticals, for which virtually no published information is available. However, some clues could be deduced in specific cases such as the endocrine disruptors bisphenols, known to produce catechol moiety-containing chemicals after metabolisation (Ye et al., 2011), which could therefore contribute to iron sequestration phenomena similar to those explained for catecholamines. In any case, demonstrating this potential mechanism in particular and delving into the completely unknown ones poses a knowledge gap that research should solve in order to better understand the implications of these mediators as added threats for the infected host.

Some other phenomena reviewed here have been remarkably characterized in terms of the underlying mechanisms, as for instance happens with the aforementioned iron sequestration-based virulence improvements linked to catechol moiety-containing chemicals. Interestingly, there are also some cases of similarities in underlying mechanisms when comparing the virulence-boosting effects caused by endogenous vs exogenous chemicals, e.g. promotion of envelope/oxidative stress, activation of two-component systems, etc. which indicates the timeliness of our integrative perspective in this review. Regardless of these occasional common points, the bacterial mediators identified (i.e. iron acquisition transporters, two-component systems, specific receptors such as Ef-Tu, etc.) should be considered as potential therapeutic targets to curb the severity of certain infections at least in some contexts (e.g. situations of stress hormone release or exposure to certain medications). The validity of this idea has, in fact, been demonstrated for human adrenergic receptor antagonists, shown to abrogate the virulence-boosting effects of catecholamines in different pathogens likely by blocking the bacterial receptors that activate pathogenic-increasing responses. Additionally, when dealing with endogenous chemicals, targeting the host’s production of certain mediators acting as pathogenic boosters (such as the use of contraceptives in the case of female sex hormones) could have beneficial effects in reducing bacterial virulence and therefore improving infection outputs. These strategies should be delved into, by investigating what other bacterial receptors or endogenous pathways could be targeted to minimize the effects of other less studied compounds such as neurotransmitters, cytokines, drugs, etc.

Although, as mentioned above, many drugs of generalized use (opioids, benzodiazepines, catecholamine inotropes, etc.) display pathogenic-enhancing effects, an analysis of these potentials in several other drugs has not (or only scarcely) been approached, posing a knowledge gap that needs filling. In this regard, and in accordance with what has been stated about the iron sequestration mechanisms underlying catecholamine-driven virulence modulations, any drug containing a catechol moiety should be seen as a potential enhancer for pathogenic success through this pathway. Thus, dipivefrine (used for glaucoma treatment) or some pharmaceuticals usually administered to Parkinson’s patients (such as L-DOPA, carbidopa and others), which have never been specifically analyzed in terms of their potential influence on bacterial behavior should be considered in this regard. These ideas should also be extended to pollutants that contain catechol or similar moieties in their structures (IARC, 1999) and for which no information regarding their capacity to modulate virulence is available, thereby posing a research field worth being urgently opened.

Moreover, other non-catechol drugs generally administered to the population such as anti-depressants, antihypertensive medications, drugs for diabetes or hypercholesterolemia, analgesics, anxiolytics, anti-inflammatory drugs, etc. should also be approached given the noteworthy exposure (even on a daily basis in many cases) that many people have to them (Verdin, 2026), and given the lack of information regarding their potential to affect bacterial pathogenesis. The same reasoning could be applied to other relevant pollutants to which we are exposed very frequently (e.g. carbamates, pyrethroids, polychlorinated biphenyls, dioxins, pesticides such as DDT, etc.), which have been barely investigated in terms of their potentials for bacterial virulence modulation (Shetty et al., 2023). Finally, dissection of some virulence-boosting challenges (e.g. tobacco smoke, e-cigarette vapor) that contain a plethora of different compounds (benzopyrene, nitrosamines, formaldehyde, tar, etc.), should be approached in order to identify the specific chemicals, besides nicotine and the oxidative stress itself, that effectively influence virulence. This could be the key to find therapeutic targets in pathogen pathways finally acting as brakes for their virulence.

In conclusion, this review highlights the fact that there are many knowledge gaps in the topic, but also several clues that could, potentially, be exploitable from the therapeutic perspective. Thus, research should continue investigating what chemicals we produce/come into contact with and which receptors/pathways may contribute to aggravate infections by enhancing bacterial virulence, so as to identify neglected day-to-day threats and to design appropriate preventive/therapeutic strategies accordingly. Gaining data into this field could help combat one of the greatest adversaries facing public health in the 21^st^ century, as are bacterial infectious diseases and their often-associated antibiotic resistance phenomena.
